# Bridging therapy versus direct mechanical thrombectomy in acute ischemic stroke: an updated meta-analysis of real-world evidence

**DOI:** 10.3389/fmed.2025.1731626

**Published:** 2026-01-09

**Authors:** Yinsheng Huang, Xiuping Wang, Gaoyang Sheng, Xujian Miao

**Affiliations:** Department of Neurology, Longyou County People’s Hospital, Quzhou, Zhejiang, China

**Keywords:** acute ischemic stroke, bridging therapy, intravenous thrombolysis, mechanical thrombectomy, meta-analysis

## Abstract

While randomized controlled trials (RCTs) have compared bridging therapy (BT: IV thrombolysis prior to mechanical thrombectomy) with direct mechanical thrombectomy (dMT) in patients with acute ischemic stroke (AIS), their findings are inconsistent and may not fully represent real-world clinical practice. This study provides an updated synthesis of real-world observational data comparing the safety and efficacy of BT versus dMT in AIS due to large vessel occlusion (LVO). A systematic literature search was conducted across four major databases. Non-randomized studies comparing BT with dMT in AIS patients were included. Pooled odds ratios (ORs) with 95% confidence intervals (CIs) were calculated using random-effects models for key clinical outcomes. Risk of bias was assessed using the Newcastle–Ottawa Scale, and publication bias was evaluated through funnel plot symmetry and Egger’s test. Thirty-one observational studies involving 93,297 patients (41,393 BT; 47,960 dMT) were included. BT was associated with significantly higher odds of excellent [modified Rankin Scale (mRS) 0–1; OR = 1.51, 95%CI: 1.30–1.77] and favorable (mRS 0–2; OR = 1.44, 95% CI: 1.29–1.61) recovery at 90 days, greater rates of successful reperfusion (TICI 2b/3; OR = 1.23, 95%CI: 1.09–1.39), and lower 90-day mortality (OR = 0.61, 95% CI: 0.52–0.71) compared with dMT. No significant differences were found in rates of symptomatic intracranial hemorrhage. Sensitivity analyses and publication bias assessments supported the robustness of these findings. Meta-regression identified baseline ASPECTS, NIHSS score, and several workflow intervals as significant predictors of outcome variability. These results support BT’s continued relevance in routine AIS care.

**Systematic review registration:**

PROSPERO no: CRD420251119894.

## 1 Introduction

Acute ischemic stroke (AIS) caused by large vessel occlusion (LVO) remains a leading cause of death and disability worldwide, with mechanical thrombectomy (MT) having emerged as the cornerstone of reperfusion therapy for eligible patients (1). In clinical practice, many patients who qualify for MT also meet criteria for intravenous thrombolysis (IVT), leading to the use of “bridging therapy” (BT)—IVT administration followed by MT—as a standard strategy recommended by international guidelines (2). The rationale behind BT lies in its potential to promote earlier recanalization, alter clot properties to facilitate mechanical retrieval, and address distal emboli that may be inaccessible to thrombectomy (3).

However, the clinical utility of BT compared with direct MT (dMT) has been the subject of considerable debate. While several observational studies have historically suggested that BT may be associated with better functional outcomes (4–8), randomized controlled trials (RCTs) designed to answer this question have yielded mixed results. To date, six RCTs have compared BT and dMT head-to-head, with most showing comparable efficacy and safety profiles between the two strategies (9–14). These trials have been synthesized in multiple recent meta-analyses (15, 16), which have increasingly guided clinical practice and policy recommendations.

Yet, RCTs, by design, enroll carefully selected patient populations under controlled conditions and may not fully reflect the complexity, comorbidities, and variations in care delivery present in real-world settings. Moreover, the existing RCTs differ considerably in design, population demographics, IVT protocols (e.g., alteplase vs. tenecteplase), and timing metrics, and some were underpowered or terminated early, further limiting their external validity. Real-world data—derived from observational studies, registries, and routine clinical practice—can provide valuable complementary insights, particularly concerning safety outcomes and generalizability.

A recent review by Qin et al. (17) synthesized real-world evidence from 12 registry-based studies, supporting the benefit of BT over dMT in terms of functional outcomes and mortality without a significant increase in hemorrhagic risk. However, the field has rapidly evolved, with new large-scale observational studies published in the interim. Furthermore, prior reviews often relied on unadjusted data or pooled estimates without fully addressing potential confounding through statistical matching or regression adjustment.

In this context, we conducted an updated meta-analysis focusing exclusively on non-randomized real-world studies comparing BT versus dMT in patients with AIS due to LVO. Our primary objective was to re-evaluate the safety and efficacy of BT using the most recent and methodologically robust observational evidence. Importantly, by limiting our analysis to non-RCT data, we aimed to capture a more accurate depiction of treatment performance in routine clinical practice and later compare these findings with the aggregated evidence from the six existing RCTs in the discussion.

## 2 Materials and methods

### 2.1 Protocol and reporting standards

This meta-analysis was conducted in accordance with the Preferred Reporting Items for Systematic Reviews and Meta-Analyses (PRISMA) guidelines (18) and followed methodological recommendations outlined by the Meta-analysis Of Observational Studies in Epidemiology (MOOSE) statement (19). The protocol was registered with the International Prospective Register of Systematic Reviews (PROSPERO; CRD420251119894).

### 2.2 Literature search strategy

A comprehensive literature search was performed across PubMed, Scopus, Web of Science, and Google Scholar from database inception through June 8th, 2025, without restrictions on language or publication status. The search strategy combined controlled vocabulary and free-text terms related to “acute ischemic stroke,” “intravenous thrombolysis,” “bridging therapy,” “mechanical thrombectomy,” and “endovascular therapy.” An experienced medical librarian validated the final strategy. The complete search syntax for each database is available in Supplementary Table S1. Additionally, we manually screened the reference lists of relevant articles and reviews for additional eligible studies (20).

### 2.3 Eligibility criteria

We included non-randomized studies (prospective or retrospective cohort studies, registries, or non-randomized interventional studies) that directly compared bridging therapy (IV thrombolysis prior to mechanical thrombectomy or endovascular therapy) with direct mechanical thrombectomy (dMT) in adult patients with acute ischemic stroke. Studies were eligible if they reported on at least one of the following outcomes: functional recovery [modified Rankin Scale (mRS)], successful reperfusion (Thrombolysis in Cerebral Infarction Score “TICI” 2b/3), symptomatic or asymptomatic intracerebral hemorrhage (sICH/aICH), or mortality at 90 days. Randomized controlled trials, review articles, conference abstracts without full texts, animal studies, and studies not specifying IVT status prior to MT were excluded. Duplicate reports and post-hoc analyses of randomized trials were also excluded.

### 2.4 Study selection

After deduplication in EndNote (Clarivate Analytics), two reviewers independently screened titles and abstracts. Full texts of potentially relevant articles were retrieved and reviewed for inclusion. Discrepancies were resolved through discussion or by the senior reviewer.

### 2.5 Data extraction

Data were independently extracted by two reviewers using a standardized, pilot-tested form. The following information was collected: first author, publication year, country, study design, sample size, number of patients in each group (BT and dMT), baseline characteristics (age, sex, NIHSS, ASPECTS), timing metrics (e.g., onset-to-groin puncture time), adjustment methods for confounding (e.g., propensity score matching or regression), and outcomes of interest. When numerical data were not directly reported, estimates were extracted from plots or calculated from available data. Authors were contacted for missing or unclear data as needed.

### 2.6 Risk of bias assessment

The methodological quality of included studies was appraised using the Newcastle–Ottawa Scale (NOS) for cohort studies (21). This tool evaluates three domains: selection, comparability, and outcome assessment, with scores ranging from 0 to 9. Studies scoring 7–9 were considered high quality, 5–6 moderate quality, and <5 low quality. All assessments were conducted independently by two reviewers, with disagreements resolved by consensus.

### 2.7 Statistical analysis

All analyses were performed using STATA version 18 (StataCorp LLC, College Station, TX). Pooled estimates were calculated using a random-effects model (DerSimonian and Laird method) due to expected between-study variability. The primary effect measure was the odds ratio (OR) with 95% confidence intervals (CI) for binary outcomes. Mean differences (MD) were used for continuous baseline variables. Statistical heterogeneity was assessed using the I^2^ statistic, with thresholds of 25, 50, and 75% representing low, moderate, and high heterogeneity, respectively. A *p*-value <0.10 for the Q test was considered significant for heterogeneity. Leave-one-out sensitivity analyses were conducted for all primary outcomes to test the robustness of pooled estimates. Potential publication bias was assessed visually using funnel plots and quantitatively via Egger’s regression test, with *p* < 0.05 suggesting significant asymmetry.

To explore potential sources of heterogeneity and identify study-level factors associated with variation in treatment effects, we performed meta-regression analyses for all reported outcomes. Univariable random-effects meta-regression models were constructed using the restricted maximum likelihood (REML) estimator. The following covariates were examined based on availability across studies: demographic variables (age, sex), vascular risk factors (hypertension, diabetes, atrial fibrillation, smoking, hypercholesterolemia, dyslipidemia, prior stroke), imaging and occlusion characteristics (ASPECTS, occlusion site including ICA, M1, or M2), medication history (anticoagulant or antiplatelet use), and workflow or timing metrics (onset-to-door, door-to-groin puncture, onset-to-recanalization, groin-to-revascularization, door-to-revascularization, onset-to-groin, onset-to-imaging, and imaging-to-groin). Regression coefficients, 95% confidence intervals, and *p*-values were reported.

## 3 Results

### 3.1 Literature search results

**Figure 1:**
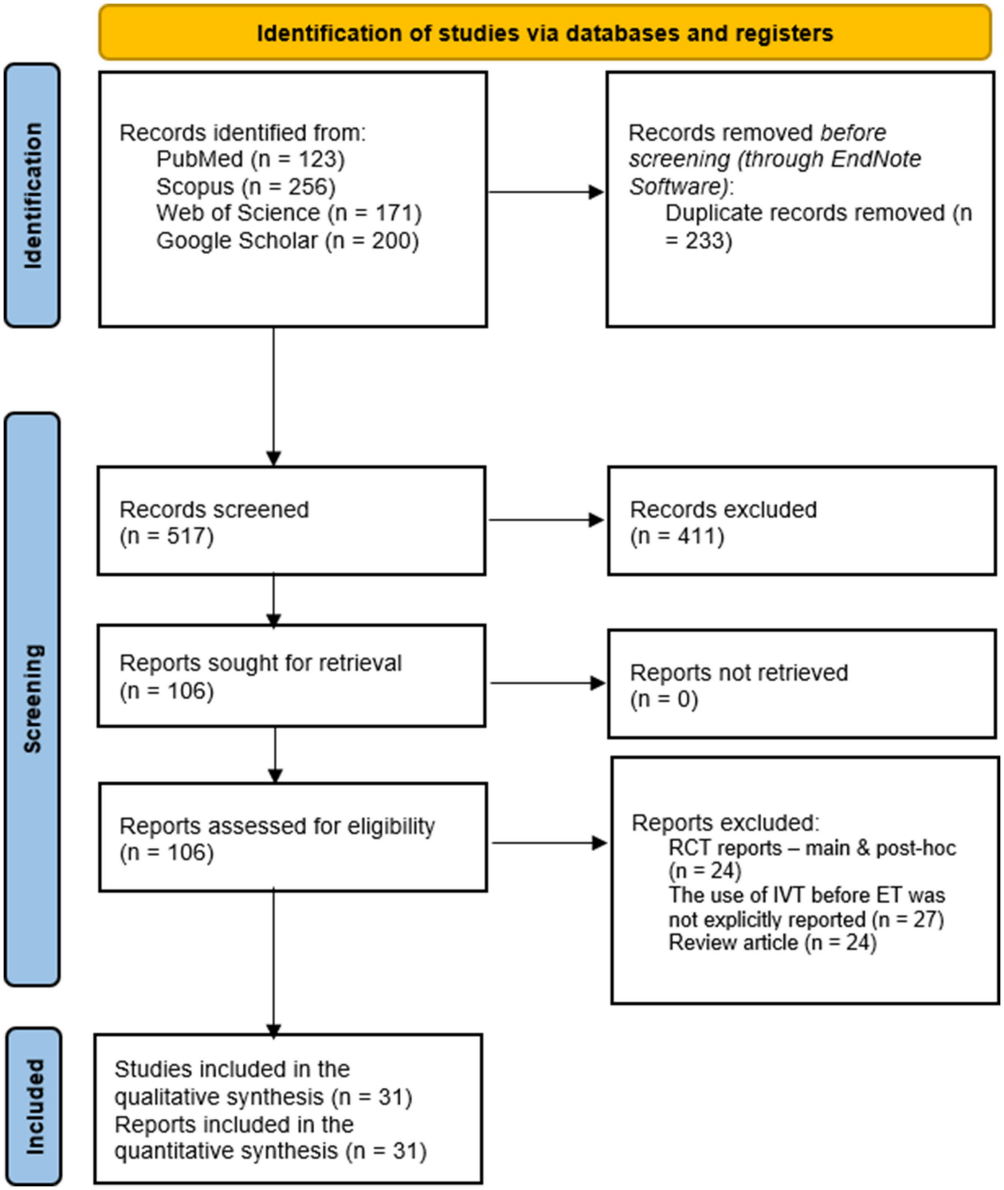
A PRISMA flow diagram showing the results of the database search.

The results of the literature search and screening processes are illustrated in Figure 1. The literature search identified 750 reports, of which 233 duplicates were removed through Endnote, and 517 citations were screened. Only 106 reports were retrieved for full text screening, of which 75 were excluded either for not explicitly reporting BT use or IVT use before endovascular therapy (27 reports), being randomized trials (24 main and post-hoc reports), or review articles (24 reports). A complete list of excluded articles can be found in Supplementary Table S2. Finally, 31 articles were eligible for analysis (4–8, 22–47).

### 3.2 Baseline characteristics of included studies

A summary of the baseline characteristics of included studies can be found in Table 1. Overall, most evidence came from prospective cohort studies (16 reports, 51.62%), followed by retrospective cohorts and registry-based studies (14 reports, 45.16%), with a single non-randomized study of intervention (3.23%). Most research was done in the United Stated (7 studies, 22.58%), followed by China (6 studies, 19.35%), and France (5 studies, 16.13%), respectively. Most studies used propensity-score matching or regression to adjust for confounding (24 studies, 77.42%). A total of 93,297 stroke patients were examined, of whom 41,393 cases underwent BT and 47,960 cases underwent dMT. The BT group was associated with a lower age (MD = −1.18 years) and higher male frequency (2.125%) than the dMT group. The NIHSS score at admission was 16.03 in the BT group and 15.79 in the dMT group (MD = 0.26). The ASPECTS score at admission was 7.82 in the BT group and 7.74 in the dMT group (MD = 0.075). The mean onset-to-groin puncture time was slightly lower in the BT group than the dMT group (MD = −54.54).

**Table 1:** Baseline characteristics of real-world studies comparing BT to dMT in acute stroke settings.

Author (YOP)	Design	Country	Sample		Age		Male (%)		ICA (%)		M1 (%)		M2 (%)		Confounding adjustment (regression)
			BT	dMT	BT	dMT	BT	dMT	BT	dMT	BT	dMT	BT	dMT	
Chang et al. (2020) (24)	PC	USA	87	83	68.4	69.8	46	38.5	28.5	32	55	50.5	17	16	Yes
Da Ros (2021) (56)	RC	USA	1,226	1,669	69.23	69.9	45.9	45.8	–	–	–	–	–	–	Yes
Derraz et al. (2023) (25)	RC	USA	603	660	66.8	68.4	56.6	55.6	27.5	24.1	53.2	53.6	4.1	6.5	Yes
Di Maria et al. (2018) (5)	PC	France	976	531	67.2	67.6	54.3	51.2	17.2	21.3	51.1	50.7	13.2	12.2	Yes
Dicpinigaitis et al. (2022) (26)	RC	USA	19,735	28,790	68.9	69.7	49.1	47	–	–	–	–	–	–	Yes
El Malky et al. (2022) (27)	RC	Egypt	150	51	–	–	–	–	16	11.8	53.3	64.7	–	–	Yes
Faizy et al. (2022) (28)	RC	USA	365	352	75	76	52	45	18	22	59	60	23	18	Yes
Fang et al. (2022) (29)	pc	China	257	593	–	–	–	–	–	–	–	–	–	–	Yes
Ferrigno et al. (2018) (6)	PC	France	348	137	66.3	67.1	46	44.5	–	–	–	–	–	–	Yes
Gong et al. (2019) (31)	RC	China	42	31	69	71	35.71	51.61	–	–	–	–	–	–	Yes
Guedin et al. (2015) (32)	PC	France	28	40	69.2	64.6	39.3	37.5	–	–	–	–	–	–	No
Guo et al. (2024) (33)	RC	China	119	529	62	65	74.8	74.6	–	–	–	–	–	–	Yes
Huu An et al. (2022) (34)	PC	Vietnam	30	30	66.5	64	70	60	40	33.3	50	60	10	6.7	No
Kaesmacher et al. (2018) (35)	RC	Germany	160	79	69.8	73.3	45.6	45.6	–	–	–	–	–	–	Yes
Kurminas et al. (2020) (37)	PC	Italy	38	65	67.1	68.4	42.1	40	–	–	–	–	–	–	No
Le Floch et al. (2023) (7)	RC	France	570	562	–	–	–	–	–	–	–	–	–	–	Yes
Maier et al. (2017) (39)	PC	Germany	81	28	75	76	59.4	42.9	–	–	–	–	–	–	No
Molad et al. (2023) (40)	RC	Israel	195	213	71.4	68.9	53.25	48.3	20.3	24.7	53.25	48.35	19.3	14.95	No
Seetge et al. (2024) (42)	PC	Hungary	51	31	67	72	47.1	35.5	–	–	–	–	–	–	Yes
Smith et al. (2006) (44)	NRSI	USA	30	81	65.4	66.5	43.3	43.2	–	–	–	–	–	–	No
Tong et al. (2021) (45)	PC	China	426	600	64	66	62.4	65.3	26.1	28.2	45.1	39.8	9.4	9	Yes
Wang et al. (2017) (46)	RC	China	138	138	67	67	56.5	55.1	35.5	42.7	60.1	50	4.3	7.2	Yes
Weber et al. (2017) (47)	RC	Germany	105	145	60.2	69.3	49.5	53.8	18.1	20.7	45.7	35.9	19	15.9	No
Smith et al. (2022) (43)	RC	USA	10,548	5,284	70	74	50.9	47.8	14.3	14.7	49.1	49.5	20.2	19.1	Yes
Ahmed et al. (2021) (4)	PC	EU, Norway, Iceland	3,944		72 (62–80)		50.1		–	–	–	–	–	–	Yes
Casetta et al. (2019) (22)	PC	Italy	635	513	67.6	68.8	49.3	48.9	–	–	48.8	44.8	14.2	12.5	Yes
Chalos et al. (2019) (23)	PC	Netherlands	1,161	324	70	72	54	53	6.4	3.9	58	61	13	11	Yes
Geng et al. (2021) (30)	RC	China	2069	5,605	68	67	60	60	–	–	–	–	–	–	Yes
Leker et al. (2018) (38)	PC	Israel	159	111	68.1	67.4	57	52.25	–	–	–	–	–	–	Yes
Minnerup et al. (2016) (8)	PC	France	603	504	68.3	68.7	50.4	48	32.8	34.5	44.4	37.3	10.3	13.3	Yes
Park et al. (2017) (41)	PC	Korea	458	181	68	69	57	57	40	39	–	–	–	–	Yes

YOP, year of publication; PC, prospective cohort; RC, retrospective cohort; EU, European union; USA, United States of America; NRSI, non-randomized study of intervention. Age is presented as mean.

### 3.3 Methodological quality

A summary of the methodological quality of included studies using the NOS scale can be found in Table 2. Overall, a total of 24 studies had good methodological quality, while 7 studies had fair quality.

**Table 2:** A summary of the methodological quality of included studies assessed by the Newcastle Ottawa Scale for cohort studies.

Author (YOP)	Selection				Comparability	Outcome			Overall Rating
	D1	D2	D3	D4	D5	D6	D7	D8	
Chang et al. (2020) (24)	*	*	*	*	*	*	*	*	Good
Da Ros (2021) (56)	*	*	*	*	*	*	*	*	Good
Derraz et al. (2023) (25)	*	*	*	*	*	*	*	*	Good
Di Maria et al. (2018) (5)	*	*	*	*	*	*	*	*	Good
Dicpinigaitis et al. (2022) (26)	*	*	*	*	*	*	*	*	Good
El Malky et al. (2022) (27)	*	*	*	*	*	*	*	*	Good
Faizy et al. (2022) (28)	*	*	*	*	*	*	*	*	Good
Fang et al. (2022) (29)	*	*	*	*	*	*	*	*	Good
Ferrigno et al. (2018) (6)	*	*	*	*	*	*	*	*	Good
Gong et al. (2019) (31)	*	*	*	*	*	*	*	*	Good
Guedin et al. (2015) (32)	*	*	*	*	–	*	*	*	Fair
Guo et al. (2024) (33)	*	*	*	*	*	*	*	*	Good
Huu An et al. (2022) (34)	*	*	*	*	–	*	*	*	Fair
Kaesmacher et al. (2018) (35)	*	*	*	*	*	*	*	*	RC
Kurminas et al. (2020) (37)	*	*	*	*	–	*	*	*	Fair
Le Floch et al. (2023) (7)	*	*	*	*	*	*	*	*	Good
Maier et al. (2017) (39)	*	*	*	*	–	*	*	*	Fair
Molad et al. (2023) (40)	*	*	*	*	–	*	*	*	Fair
Seetge et al. (2024) (42)	*	*	*	*	*	*	*	*	Good
Smith et al. (2006) (44)	*	*	*	*	–	*	*	*	Fair
Tong et al. (2021) (45)	*	*	*	*	*	*	*	*	Good
Wang et al. (2017) (46)	*	*	*	*	*	*	*	*	Good
Weber et al. (2017) (47)	*	*	*	*	–	*	*	*	Fair
Smith et al. (2022) (43)	*	*	*	*	*	*	*	*	RC
Ahmed et al. (2021) (4)	*	*	*	*	*	*	*	*	Good
Casetta et al. (2019) (22)	*	*	*	*	*	*	*	*	Good
Chalos et al. (2019) (23)	*	*	*	*	*	*	*	*	Good
Geng et al. (2021) (30)	*	*	*	*	*	*	*	*	Good
Leker et al. (2018) (38)	*	*	*	*	*	*	*	*	Good
Minnerup et al. (2016) (8)	*	*	*	*	*	*	*	*	Good
Park et al. (2017) (41)	*	*	*	*	*	*	*	*	Good

YOP, year of publication. D1, Representativeness of the exposed cohort; D2, selection of the non-exposed cohort; D3, ascertainment of exposure; D4, demonstration that outcome of interest was not present at start of study; D5, Comparability of cohorts on the basis of the design or analysis; D6, Assessment of outcome; D7, was follow-up long enough for outcomes to occur; D8, Adequacy of follow up of cohorts.

### 3.4 Excellent functional recovery (mRS 0–1) at 90 days

**Figure 2:**
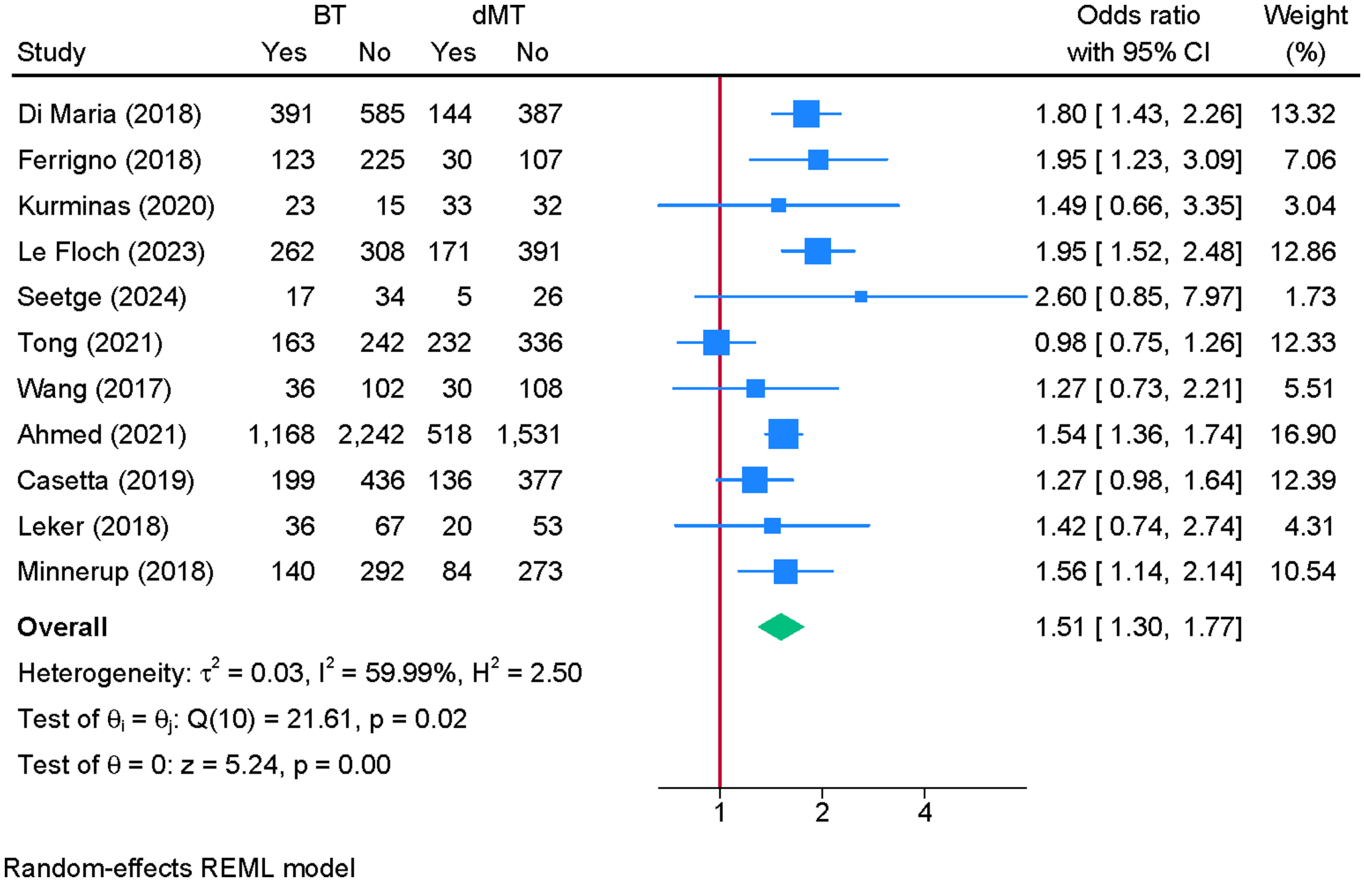
Forest plot showing the difference in excellent recovery between bridging therapy and direct mechanical thrombectomy at 90 days.

Eleven studies were eligible for meta-analysis. BT was associated with a significantly greater odds of excellent functional recovery compared to dMT (OR = 1.51; 95% CI: 1.30, 1.77) (Figure 2). Although heterogeneity was moderate (I^2^ = 59.99%, *p* = 0.02), the leave-one-out sensitivity analysis showed no change in the reported estimate (Supplementary Figure S1). The funnel plot showed no significant deviation (Supplementary Figure S2), and the Egger’s regression test showed no risk of publication bias (*p* = 0.680).

### 3.5 Favorable functional recovery (mRS 0–2) at 90 days

**Figure 3:**
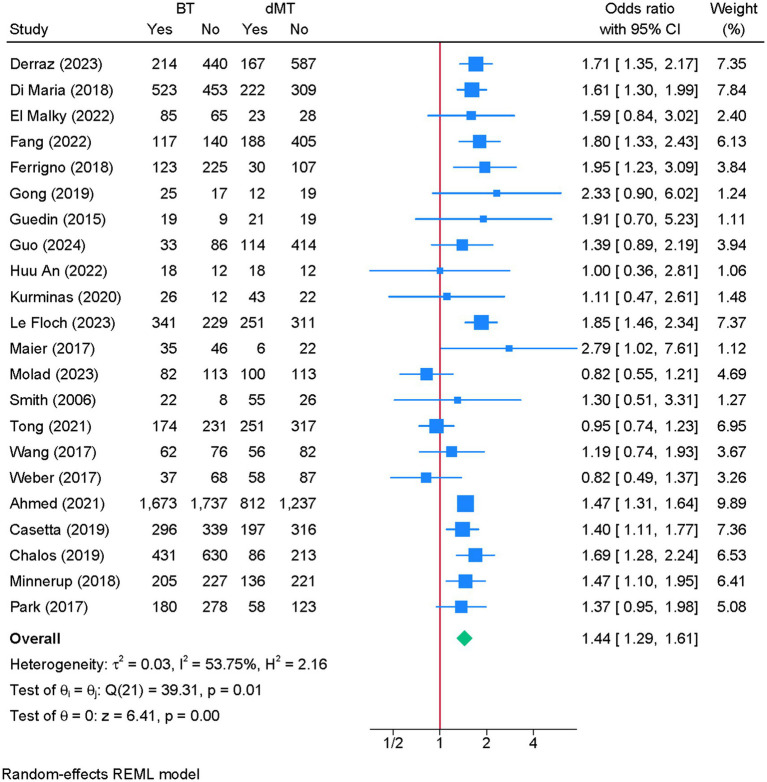
Forest plot showing the difference in favorable recovery between bridging therapy and direct mechanical thrombectomy at 90 days.

Twenty-two studies were eligible for meta-analysis. BT was associated with a significantly greater odds of favorable functional recovery compared to dMT (OR = 1.44; 95% CI: 1.29, 1.61) (Figure 3). Although heterogeneity was moderate (I^2^ = 53.75%, *p* = 0.01), the leave-one-out sensitivity analysis showed no change in the reported estimate (Supplementary Figure S3). The funnel plot showed no significant deviation (Supplementary Figure S4), and the Egger’s regression test showed no risk of publication bias (*p* = 0.881).

### 3.6 Successful reperfusion (TICI 2b/3) at 90 days

**Figure 4:**
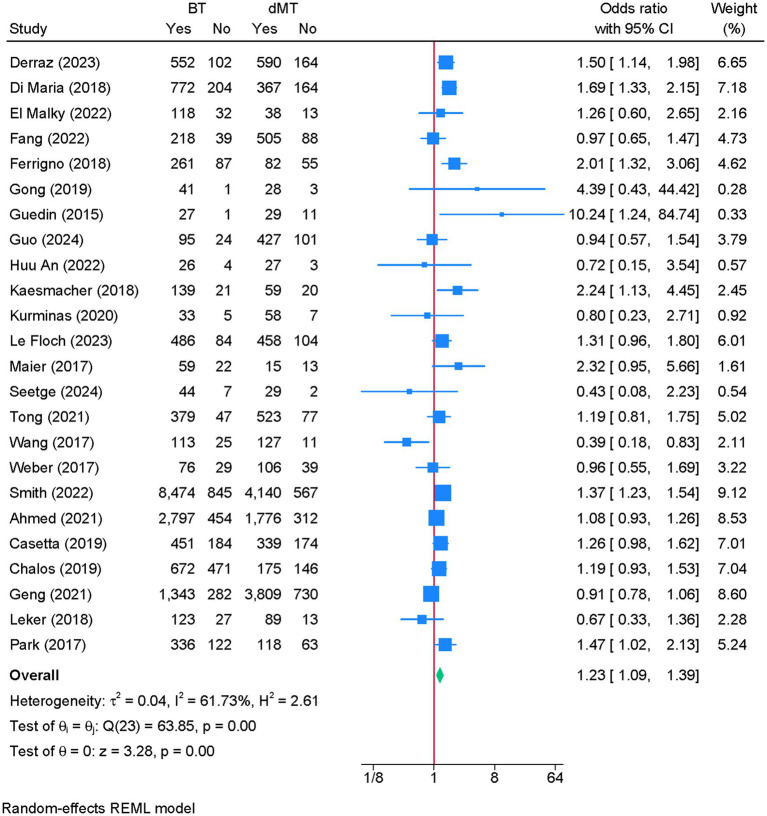
Forest plot showing the difference in successful reperfusion between bridging therapy and direct mechanical thrombectomy at 90 days.

Twenty-four studies were eligible for meta-analysis. BT was associated with a significantly greater odds of successful reperfusion compared to dMT (OR = 1.23; 95% CI: 1.09, 1.39) (Figure 4). Although heterogeneity was moderate (I^2^ = 61.73%, *p* < 0.01), the leave-one-out sensitivity analysis showed no change in the reported estimate (Supplementary Figure S5). The funnel plot showed no significant deviation (Supplementary Figure S6), and the Egger’s regression test showed no risk of publication bias (*p* = 0.986).

### 3.7 aICH at 90 days

**Figure 5:**
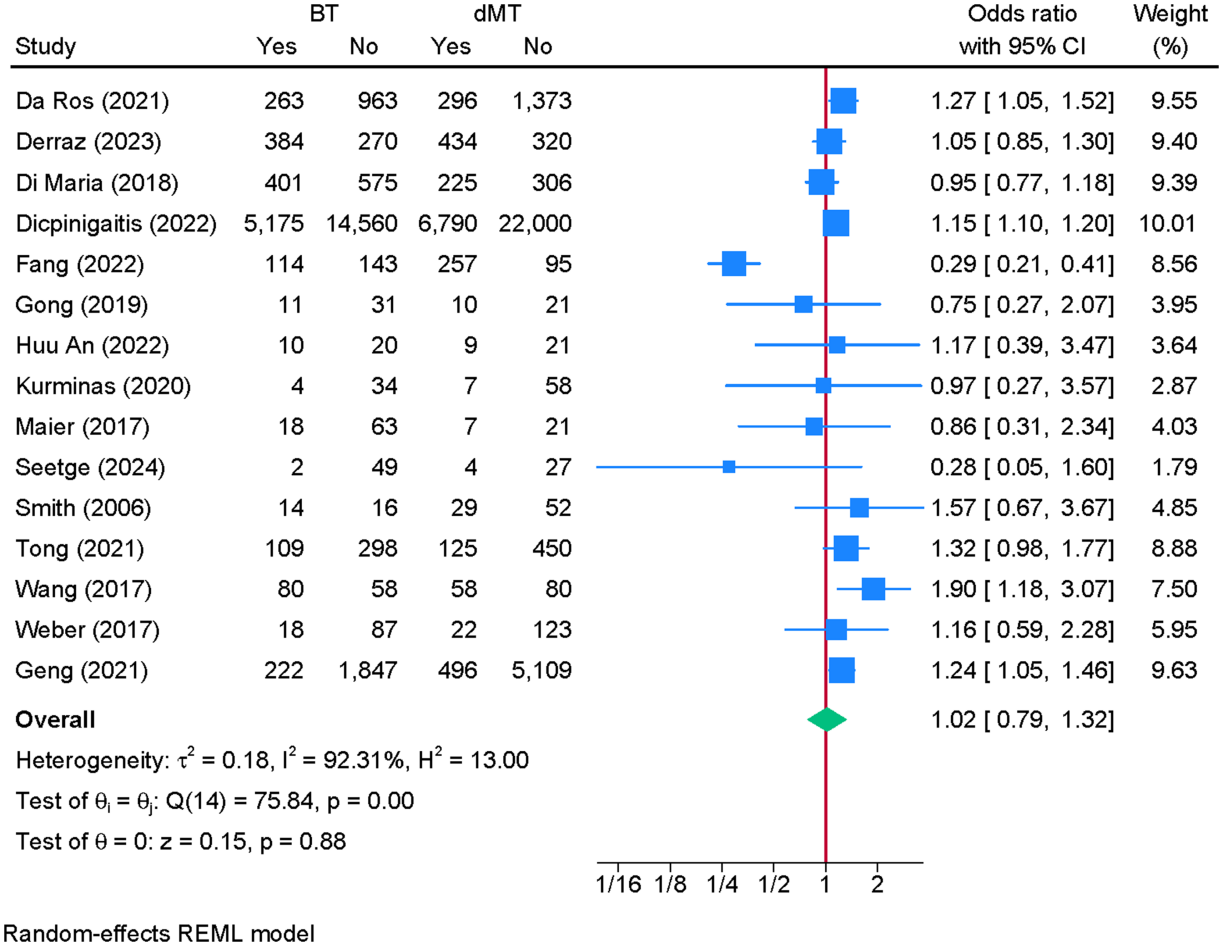
Forest plot showing the difference in the risk of any intracranial hemorrhage between bridging therapy and direct mechanical thrombectomy at 90 days.

**Figure 6:**
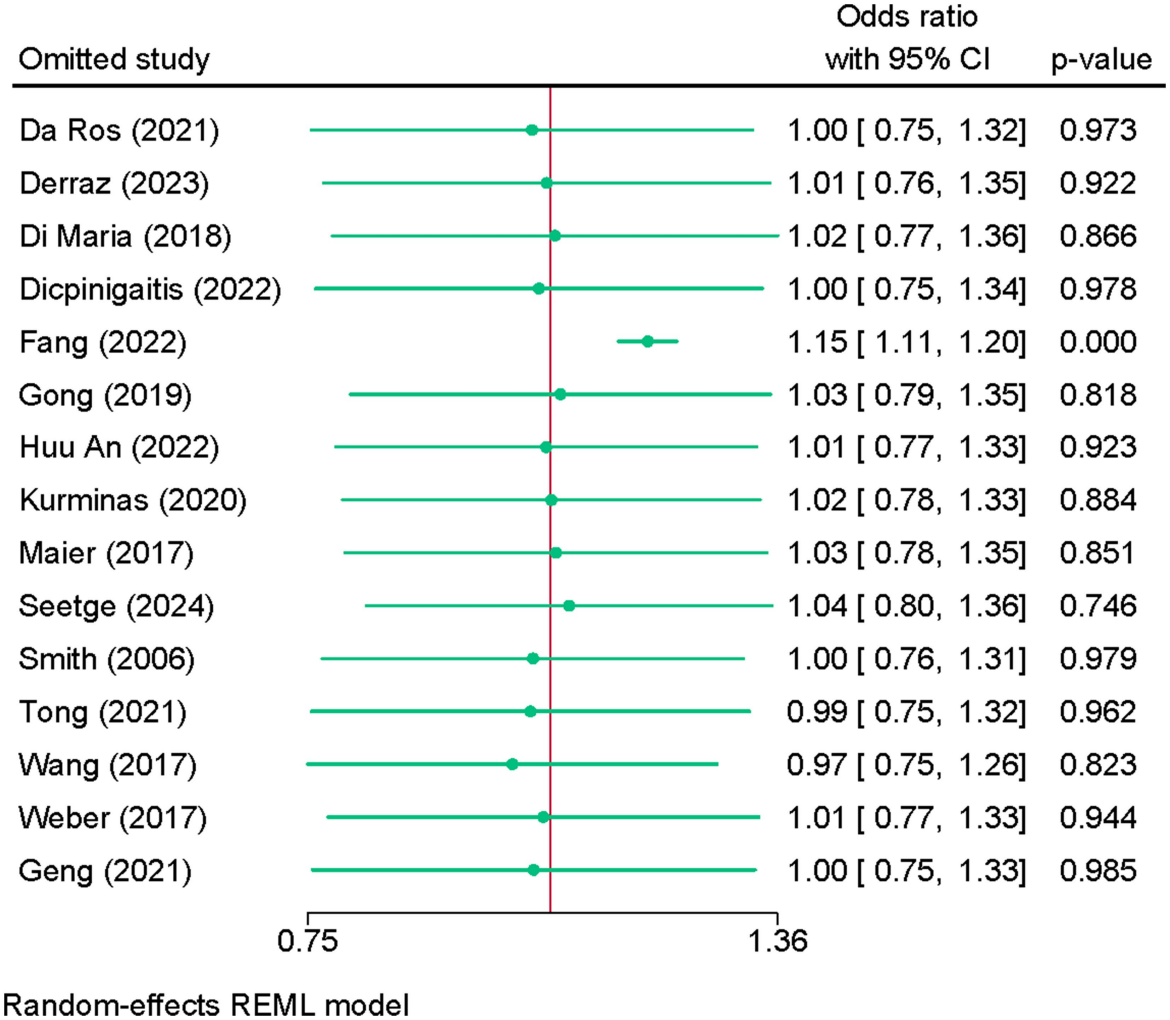
Sensitivity analysis of the difference in the risk of any intracranial hemorrhage between bridging therapy and direct mechanical thrombectomy at 90 days.

Fifteen studies were eligible for meta-analysis. No difference in the risk of aICH was noted between BT and dMT (OR = 1.02; 95% CI: 0.79, 1.32) (Figure 5). Heterogeneity was high (I^2^ = 92.31%, *p* < 0.001). The leave-one-out sensitivity analysis showed a significantly higher risk of aICH with BT vs. dMT after excluding the study of Fang et al. (29) (OR = 1.15; 95% CI: 1.11, 1.20) (Figure 6). The funnel plot showed no significant deviation (Supplementary Figure S7), and the Egger’s regression test showed no risk of publication bias (*p* = 0.494).

### 3.8 sICH at 90 days

**Figure 7:**
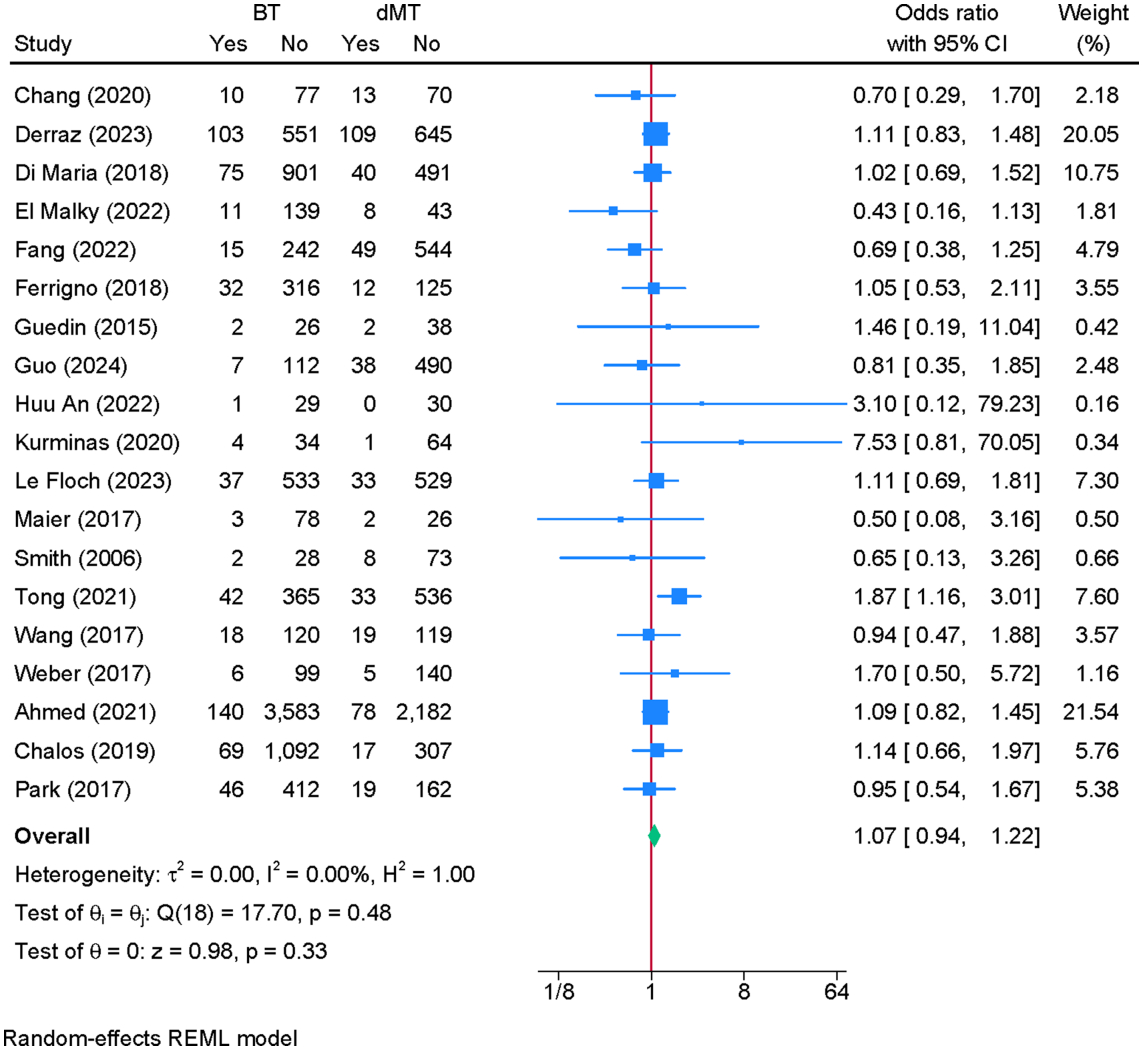
Forest plot showing the difference in the risk of symptomatic intracranial hemorrhage between bridging therapy and direct mechanical thrombectomy at 90 days.

Nineteen studies were eligible for meta-analysis. There was no difference in the risk of sICH between BT and dMT (OR = 1.07; 95% CI: 0.94, 1.22) (Figure 7). No heterogeneity was observed (I^2^ = 0%, *p* = 0.48). The funnel plot showed no significant deviation (Supplementary Figure S8), and the Egger’s regression test showed no risk of publication bias (*p* = 0.736).

### 3.9 Mortality at 90 days

**Figure 8:**
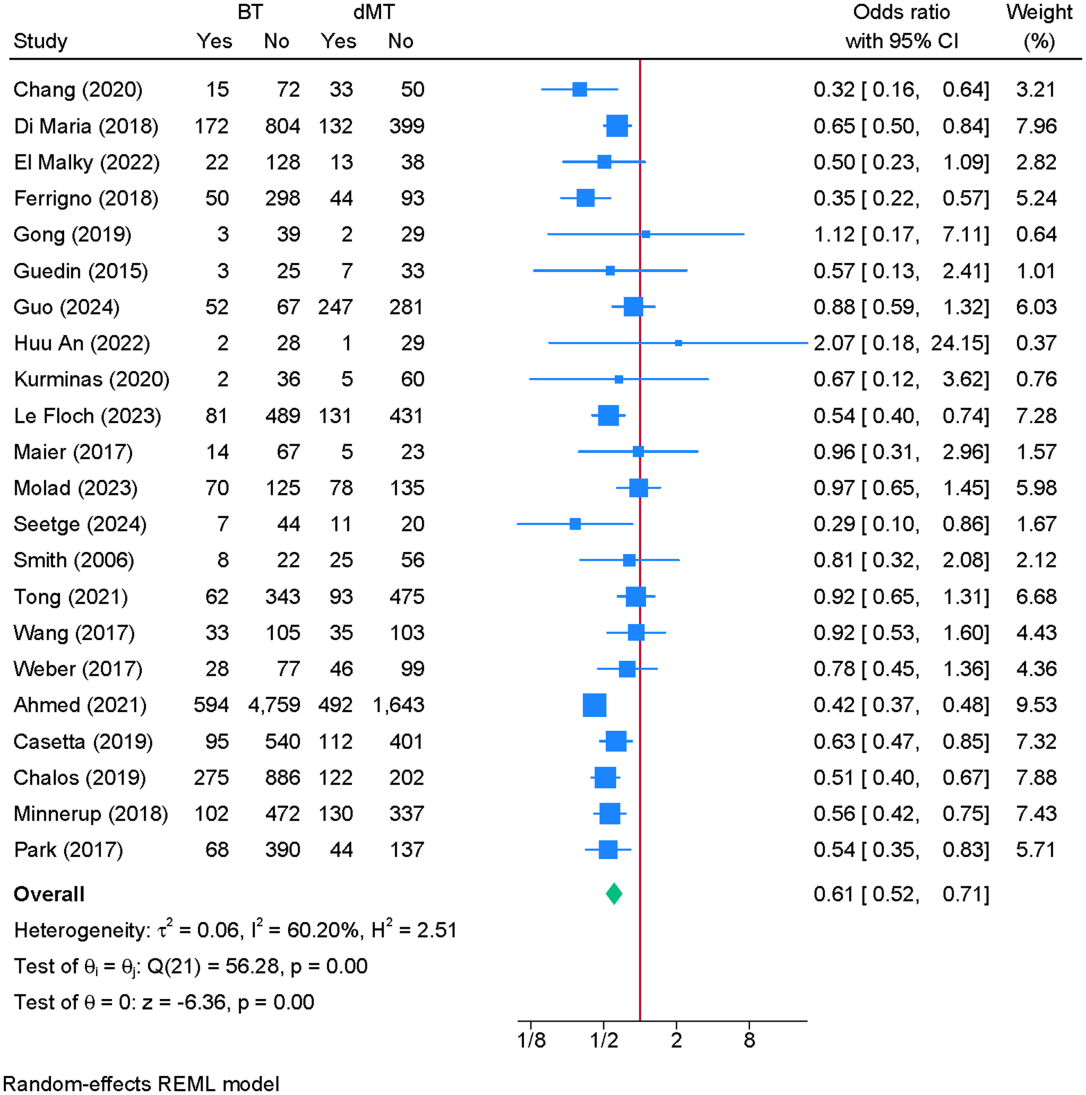
Forest plot showing the difference in the risk of death between bridging therapy and direct mechanical thrombectomy at 90 days.

Twenty-two studies were eligible for meta-analysis. BT was associated with a significantly lower risk of 90-day mortality compared to dMT (OR = 0.61; 95% CI: 0.52, 0.71) (Figure 8). Although heterogeneity was moderate (I^2^ = 60.20%, *p* < 0.01), the leave-one-out sensitivity analysis showed no change in the reported estimate (Supplementary Figure S9). The funnel plot showed no significant deviation (Supplementary Figure S10), and the Egger’s regression test showed no risk of publication bias (*p* = 0.406).

### 3.10 Meta-regression analyses

To explore sources of heterogeneity and potential effect modifiers, we conducted meta-regression analyses for all primary outcomes using study-level covariates (Table 3). For excellent functional recovery, higher baseline ASPECTS (*p* = 0.004), higher baseline NIHSS (*p* = 0.016), lower smoking rates (*p* = 0.037), lower onset-to-imaging time (*p* = 0.042), and higher percentage of male patients (*p* = 0.040) were significantly associated with greater effect estimates. For favorable recovery, antiplatelet use, ASPECTS score, and imaging-to-groin time were among the significant predictors. For sICH, male sex, hypertension, diabetes, NIHSS, ASPECTS, and onset-to-groin time showed significant associations. For mortality and reperfusion, hypertension, prior stroke, door-to-groin time, and ASPECTS were significant modifiers.

**Table 3:** A summary of the significant determinates of reported outcomes based on meta-regression analyses.

Characteristic	Coefficient	*p*-value	Low CI	High CI
Outcome: excellent functional recovery				
Male (per %)	0.0646	0.0400	0.0030	0.1262
Smoking (per %)	−0.0580	0.0370	−0.1127	−0.0034
Mean baseline NIHSS score (per point)	0.2267	0.0160	0.0417	0.4118
Mean baseline ASPECT score (per point)	1.1438	0.0040	0.3577	1.9298
Mean onset-to-imaging time (per min)	−0.0267	0.0420	−0.0525	−0.0010
Outcome: mortality				
Hypertension (per %)	0.0318	0.0130	0.0068	0.0567
Prior stroke (per %)	0.0339	0.0040	0.0106	0.0572
Mean baseline ASPECT score (per point)	−1.3557	0.0000	−2.0898	−0.6217
Outcome: sICH				
Male (per %)	−0.0659	0.0390	−0.1284	−0.0035
Hypertension (per %)	0.0439	0.0230	0.0059	0.0818
Diabetes (per %)	−0.0885	0.0210	−0.1637	−0.0134
Mean baseline NIHSS score (per point)	−0.1833	0.0420	−0.3604	−0.0063
Mean baseline ASPECT score (per point)	−1.1361	0.0280	−2.1524	−0.1199
Mean onset-to-groin time (min)	0.0037	0.0460	0.0001	0.0074
Outcome: favorable functional recovery				
Hypertension (per %)	−0.0254	0.0280	−0.0481	−0.0027
Stroke location - M1 (per %)	−0.0280	0.0480	−0.0558	−0.0002
General anesthesia (per %)	−0.0542	0.0020	−0.0887	−0.0198
Antiplatelet use (per %)	0.0420	0.0030	0.0147	0.0693
Mean baseline ASPECT score (per point)	1.2507	0.0000	0.5981	1.9034
Mean imaging-to-groin time (per min)	−0.0216	0.0300	−0.0411	−0.0021
Outcome: successful reperfusion				
Prior stroke (per %)	−0.0260	0.0370	−0.0504	−0.0016
Mean door-to-groin time (per min)	−0.0162	0.0210	−0.0299	−0.0025

sICH, symptomatic intracranial hemorrhage; CI, confidence interval. Meta-regression models additionally examined the following variables, which were not significantly associated with any of the reported outcomes: age, sex (female %), diabetes mellitus, atrial fibrillation, smoking status, hypercholesterolemia, dyslipidemia, prior stroke, internal carotid artery (ICA) occlusion rate, M2 occlusion rate, use of anticoagulant or antiplatelet therapy, and multiple workflow-related time intervals (onset-to-door, door-to-groin puncture, onset-to-recanalization, groin-to-revascularization, door-to-revascularization, onset-to-groin, and imaging-to-groin).

## 4 Discussion

This updated meta-analysis of over 93,000 real-world patients offers compelling evidence favoring the use of BT over dMT in AIS. Compared with dMT, BT was associated with significantly greater odds of achieving excellent and favorable functional outcomes at 90 days, higher rates of successful reperfusion, and lower mortality—without a corresponding increase in the risk of hemorrhagic complications. These findings are consistent with, and extend upon, prior real-world data (17) and provide critical complementary insights to those generated by RCTs (15).

Over the past few years, at least six major RCTs—DIRECT-MT (9), DEVT (10), SKIP (11), MR CLEAN-NO IV (12), SWIFT-DIRECT (13), and DIRECT-SAFE (14)—have attempted to resolve the clinical equipoise surrounding BT versus dMT in IVT-eligible patients. Pooled results from these RCTs generally support the non-inferiority of dMT for favorable functional outcomes (15, 16, 48), but their conclusions are tempered by regional heterogeneity, differences in imaging and selection protocols, and strict eligibility criteria. For example, Liu et al. (15) emphasized that while dMT met non-inferiority thresholds in some trials, others failed to demonstrate equivalence, underscoring unresolved uncertainty in specific subgroups.

In contrast, observational data—including the current study—reflect broader clinical settings, encompassing variations in hospital infrastructure, workflow logistics, and patient-level risk factors that are often underrepresented in RCTs. In this context, the term ‘real-world’ refers to several aspects of routine clinical practice that differ from the tightly controlled conditions of RCTs. These include broader and more heterogeneous inclusion criteria, encompassing patients with wider ranges of stroke severity, comorbidities, imaging profiles (including lower ASPECTS), and treatment windows. Real-world cohorts also reflect substantial variability in workflow metrics—such as onset-to-imaging, door-to-needle, and door-to-groin times—as well as differences in hospital infrastructure, transfer patterns, operator expertise, and local protocols for IVT and thrombectomy. Additionally, physician decision-making, system-level delays, and regional differences in prehospital logistics influence treatment delivery in ways not captured in trial settings. Together, these elements provide a more comprehensive and externally valid representation of how BT and dMT perform in everyday practice. Our findings align closely with those of Katsanos et al. (49), who similarly reported superior functional outcomes and reduced mortality with BT across 11,798 patients, even after adjustment for confounders. Likewise, a recent umbrella review by Campbell et al. (50) reiterated the modest yet consistent benefits of BT for functional recovery and reperfusion success, particularly in systems with efficient in-hospital workflows and minimal IVT-to-groin delay.

Importantly, this study contributes an up-to-date synthesis of real-world data at a larger scale than any previously published review (17). Compared to Waller et al. (51), who summarized older data from 2015 to 2020 and noted favorable BT outcomes in patients with anterior circulation LVOs, our analysis captures evolving practices, wider geographical representation, and improved adjustment for confounding via propensity-score matching or regression methods in 77% of included studies.

Concerns surrounding the potential risks of BT—particularly increased rates of thrombus fragmentation, distal embolization, or hemorrhagic transformation—remain important. However, in our meta-analysis, BT was not associated with increased rates of either symptomatic or asymptomatic intracerebral hemorrhage. However, this conclusion requires caution, particularly for aICH. The substantial heterogeneity among studies reporting aICH and the sensitivity finding—where exclusion of a single study shifted the effect toward significance—indicate that uncertainty remains. Differences in imaging criteria, reporting standards, and patient selection may contribute to this variability, making the true effect of BT on aICH less certain than the pooled summary suggests. This echoes the conclusions of Tsivgoulis et al. (52), who reported no excess bleeding risk with BT in their large observational cohort. Furthermore, the absence of heterogeneity for sICH across our included studies supports the robustness of this finding.

From a pathophysiological perspective, IVT may facilitate microvascular reperfusion, soften thrombus structure, and promote early partial recanalization before thrombectomy—mechanisms hypothesized in several translational studies and supported by improved TICI 2b/3 rates in both this and earlier meta-analyses (49, 53). Moreover, BT may act as a safety net in cases of failed or delayed thrombectomy, a scenario not uncommon in real-world settings but under-represented in RCTs. Additionally, intravenous thrombolysis may preferentially target the peripheral, branching projections of the clot—a phenomenon often described as the ‘finger-like thrombus’ effect—thereby reducing clot anchoring strength and improving device engagement during thrombectomy (54). IVT has also been shown to enhance microcirculatory reperfusion by dissolving distal microemboli and restoring capillary-level flow, effects that are not directly achieved by mechanical thrombectomy alone (55). Together, these mechanisms provide complementary biological pathways through which BT may improve successful reperfusion rates and functional outcomes, particularly in real-world settings where procedural delays or anatomical challenges may limit the effectiveness of dMT.

To further explore sources of heterogeneity and strengthen the interpretability of our findings, we performed meta-regression analyses across all primary outcomes using study-level covariates. Several demographics, clinical, imaging, and workflow-related characteristics were identified as significant modifiers of treatment effect (Table 3). Higher baseline ASPECTS and NIHSS scores were consistently associated with better odds of functional recovery, underscoring the importance of initial infarct size and neurological severity. Time intervals—particularly onset-to-imaging, imaging-to-groin, and door-to-groin metrics—also influenced effect estimates, highlighting the well-established impact of workflow efficiency on reperfusion success. In addition, risk-factor profiles such as hypertension, prior stroke, and smoking contributed to variations in mortality, sICH, or reperfusion outcomes. Although these associations should be interpreted cautiously given the ecological nature of study-level meta-regression, they provide important insights into factors that may shape real-world treatment performance and help explain some of the heterogeneity observed across included studies.

Still, our study is not without limitations. The non-randomized design of included studies introduces the possibility of residual confounding, although most employed statistical adjustments. The definition and adjudication of outcomes (e.g., functional independence, hemorrhage classification) varied across studies. Also, the predominance of data from high-income settings may limit generalizability to low-resource environments. Additionally, although most included studies performed statistical adjustment, the extent to which they accounted for thrombus characteristics or collateral circulation varied substantially. Several studies included imaging-based covariates such as collateral grade, mCTA collateral score, pial arterial collateral status, ASITN/SIR collateral grade, ASPECTS, and occlusion site, which partially capture thrombus burden and baseline tissue viability (see Supplementary Table S3). However, detailed thrombus morphology (e.g., clot length, clot perviousness) and standardized collateral scoring were not uniformly reported across studies. This heterogeneity may introduce residual confounding in reperfusion and functional outcomes. As such, while the majority of analyses adjusted for key clinical and imaging variables, incomplete adjustment for thrombus- and collateral-related factors remains an inherent limitation of real-world observational evidence.

Nevertheless, our findings offer a clinically meaningful perspective: in routine clinical practice, BT appears to confer modest yet consistent advantages in functional recovery and survival without added hemorrhagic risk. These real-world benefits contrast with the marginal differences reported in RCTs, raising important questions about the external validity of trial-based treatment algorithms. A structured comparison of RCT evidence versus real-world data is presented in Table 4 to illustrate these differences. Future research should prioritize individual patient data meta-analyses and explore tailored approaches to stroke triage, perhaps incorporating clot burden, collateral status, and time metrics into treatment selection.

**Table 4:** Comparative summary of RCT evidence versus real-world meta-analysis findings for bridging therapy in acute ischemic stroke.

Domain	RCTs [del Cuadra-Campos et al. (16)]	Real-world meta-analysis (current study)
Population size	2,334 patients (6 RCTs)	93,297 patients (31 observational studies)
Study design	Highly selected, strictly controlled; non-inferiority frameworks	Heterogeneous, routine clinical care; broad inclusion
Patient selection	Strict IVT eligibility; excludes posterior circulation and late-presenters	Includes diverse presentations, comorbidities, workflows; more inclusive
Baseline stroke severity (NIHSS)	Median 15–19 across trials	Wider range; often higher variability across centers
Imaging selection (ASPECTS)	Narrow ranges (typically ASPECTS 7–10)	Broader distribution; includes lower ASPECTS in some studies
Occlusion sites	Mostly ICA or M1; minimal M2	ICA, M1, M2, and mixed anterior circulation distributions
Workflow metrics	Very short needle-to-groin intervals (8–44 min); door-to-puncture tightly controlled	Highly variable (urban vs. rural, primary vs. comprehensive centers)
Intervention fidelity	Protocol-driven; minimal delays; uniform alteplase use	Real-world variation in IVT-to-MT timing, operator skill, center resources
Functional outcomes (mRS 0–2)	OR ≈ 0.93 (no difference)	OR = 1.44 favoring BT
Excellent outcomes (mRS 0–1)	Not consistently reported across RCTs	OR = 1.51 favoring BT
Reperfusion success (TICI ≥2b/3)	Slight advantage for BT (OR ≈ 0.75 for dMT vs. BT)	Significant advantage for BT (OR = 1.23)
90-day mortality	No significant difference	Lower mortality with BT (OR = 0.61)
Hemorrhagic complications	No difference in sICH	No difference in sICH; aICH uncertain due to high heterogeneity
External validity	Limited (direct-transfer to MT-capable centers only)	High; includes varied hospital systems, workflows, geographies

Data on RCTs are derived from the recent meta-analysis by del Cuadra-Campos et al. (16). AIS, acute ischemic stroke; LVO, large vessel occlusion; BT, bridging therapy; dMT, direct mechanical thrombectomy; RCT, randomized controlled trial; IVT, intravenous thrombolysis; mRS, modified Rankin Scale; TICI, Thrombolysis in Cerebral Infarction; ICA, internal carotid artery; NIHSS, National Institutes of Health Stroke Scale; ASPECTS, Alberta Stroke Program Early CT Score; EMS, emergency medical services; sICH, symptomatic intracranial hemorrhage; aICH, asymptomatic intracranial hemorrhage.

This updated meta-analysis of real-world observational data reinforces the clinical value of bridging therapy in patients with acute ischemic stroke due to large vessel occlusion. Compared to direct mechanical thrombectomy, bridging therapy was associated with significantly improved functional outcomes, higher rates of successful reperfusion, and lower mortality—without an increased risk of hemorrhagic complications. These findings highlight the continued relevance of intravenous thrombolysis as a component of acute stroke care and suggest that, in real-world settings, the benefits of bridging therapy may be more pronounced than those observed in randomized controlled trials. As treatment paradigms evolve, individualized decision-making that incorporates both trial-based evidence and real-world data will be essential to optimizing outcomes for stroke patients.

## Data Availability

The original contributions presented in the study are included in the article/Supplementary material, further inquiries can be directed to the corresponding author.

## Supplementary material

SUPPLEMENTARY FIGURE S1 Sensitivity analysis of the excellent functional recovery outcome between bridging therapy and direct mechanical thrombectomy.

SUPPLEMENTARY FIGURE S2 Funnel plot showing the risk of publication bias in the excellent functional recovery outcome.

SUPPLEMENTARY FIGURE S3 Sensitivity analysis of the favorable functional recovery outcome between bridging therapy and direct mechanical thrombectomy.

SUPPLEMENTARY FIGURE S4 Funnel plot showing the risk of publication bias in the favorable functional recovery outcome.

SUPPLEMENTARY FIGURE S5 Sensitivity analysis of successful reperfusion outcome between bridging therapy and direct mechanical thrombectomy.

SUPPLEMENTARY FIGURE S6 Funnel plot showing the risk of publication bias in the successful reperfusion outcome.

SUPPLEMENTARY FIGURE S7 Funnel plot showing the risk of publication bias in the any intracranial hemorrhage outcome.

SUPPLEMENTARY FIGURE S8 Funnel plot showing the risk of publication bias in the symptomatic intracranial hemorrhage outcome.

SUPPLEMENTARY FIGURE S9 Sensitivity analysis of mortality risk between bridging therapy and direct mechanical thrombectomy.

SUPPLEMENTARY FIGURE S10 Funnel plot showing the risk of publication bias in the mortality outcome.
