# Supplementary figures and images for: Bridging therapy versus direct mechanical thrombectomy in acute ischemic stroke: an updated meta-analysis of real-world evidence
Source: Front Med (Lausanne). 2026 Jan 9;12:1731626. doi: 10.3389/fmed.2025.1731626 (PMC12827676; doi:10.3389/fmed.2025.1731626)

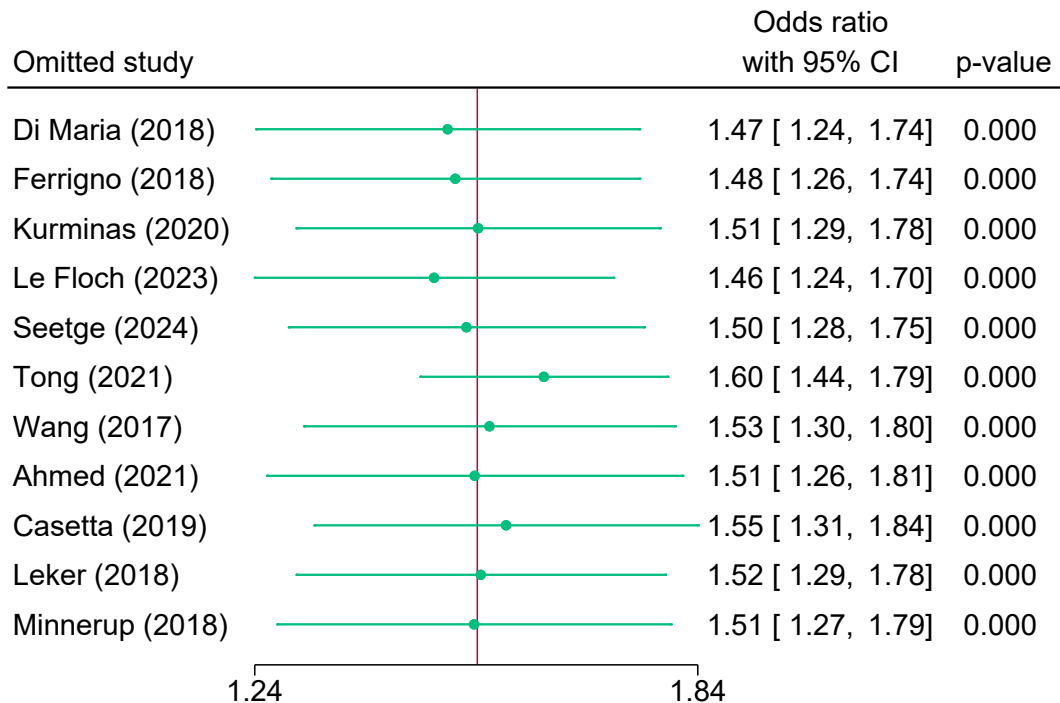

Random-effects REML model

Supplement: SUPPLEMENTARY FIGURE S1 — Sensitivity analysis of the excellent functional recovery outcome between bridging therapy and direct mechanical thrombectomy. [file Data_Sheet_1.PDF]

# Funnel plot

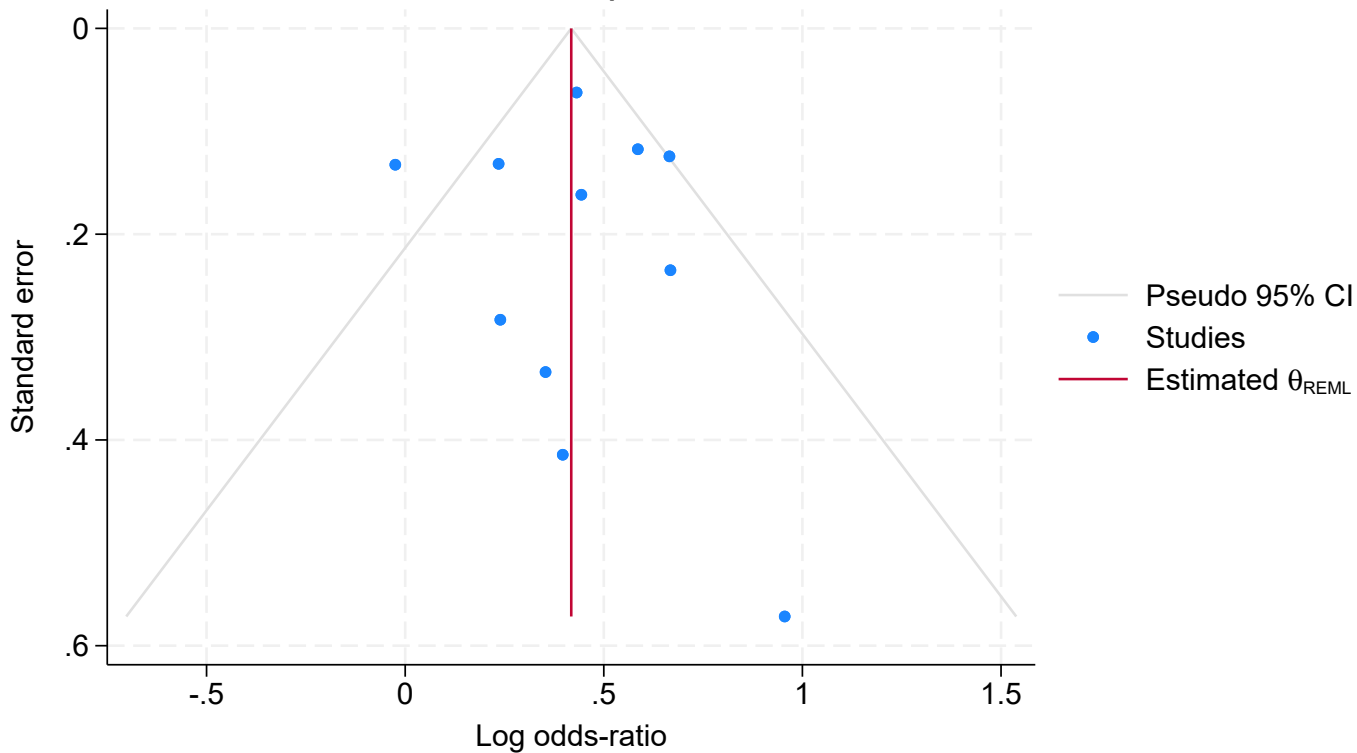

Supplement: SUPPLEMENTARY FIGURE S2 — Funnel plot showing the risk of publication bias in the excellent functional recovery outcome. [file Data_Sheet_2.PDF]

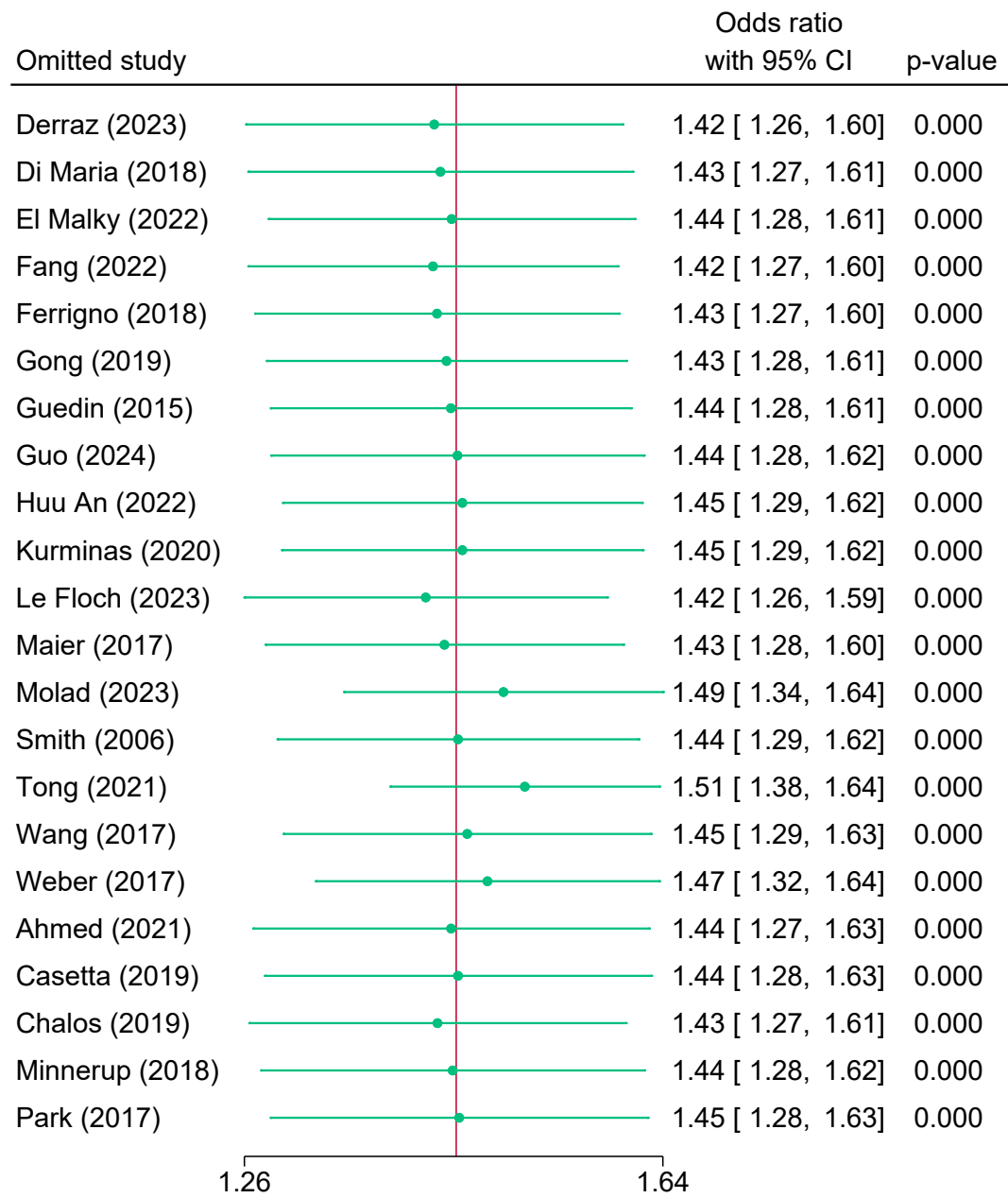

Random-effects REML model

Supplement: SUPPLEMENTARY FIGURE S3 — Sensitivity analysis of the favorable functional recovery outcome between bridging therapy and direct mechanical thrombectomy. [file Data_Sheet_3.PDF]

# Funnel plot

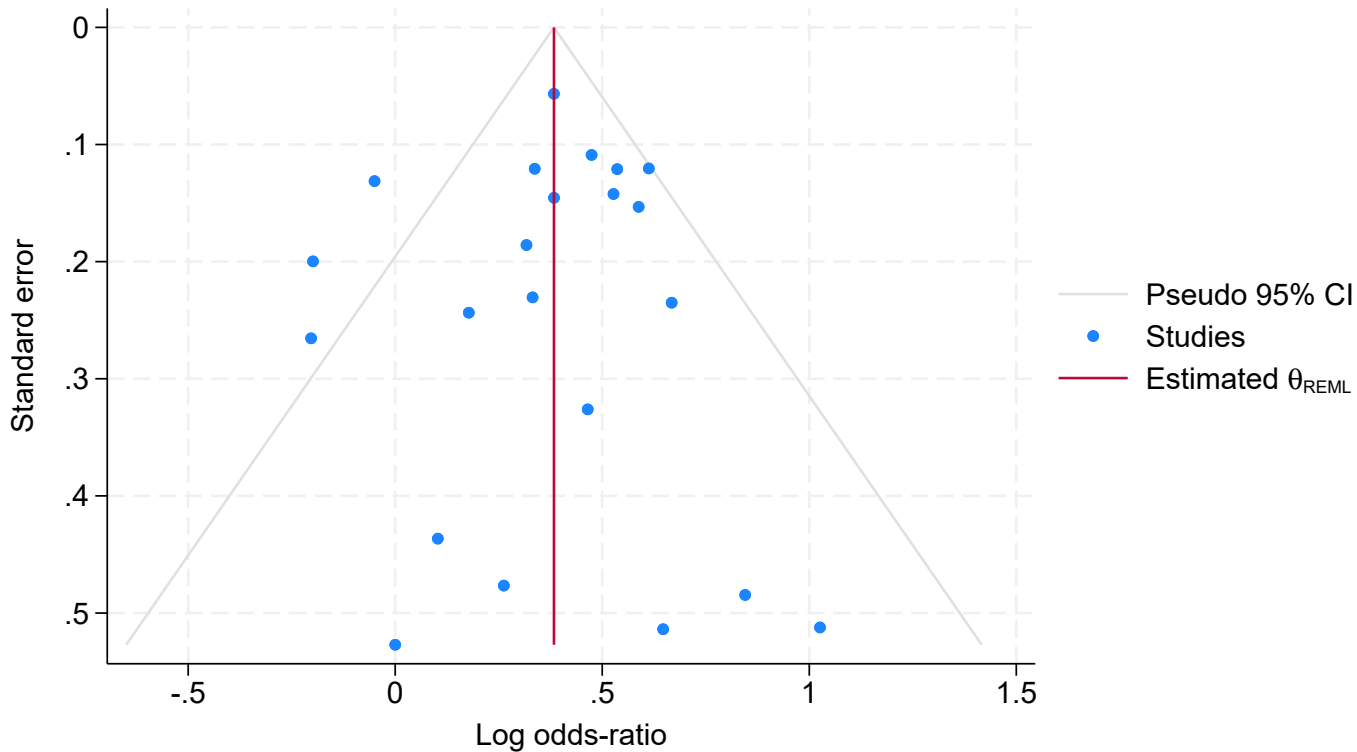

Supplement: SUPPLEMENTARY FIGURE S4 — Funnel plot showing the risk of publication bias in the favorable functional recovery outcome. [file Data_Sheet_4.PDF]

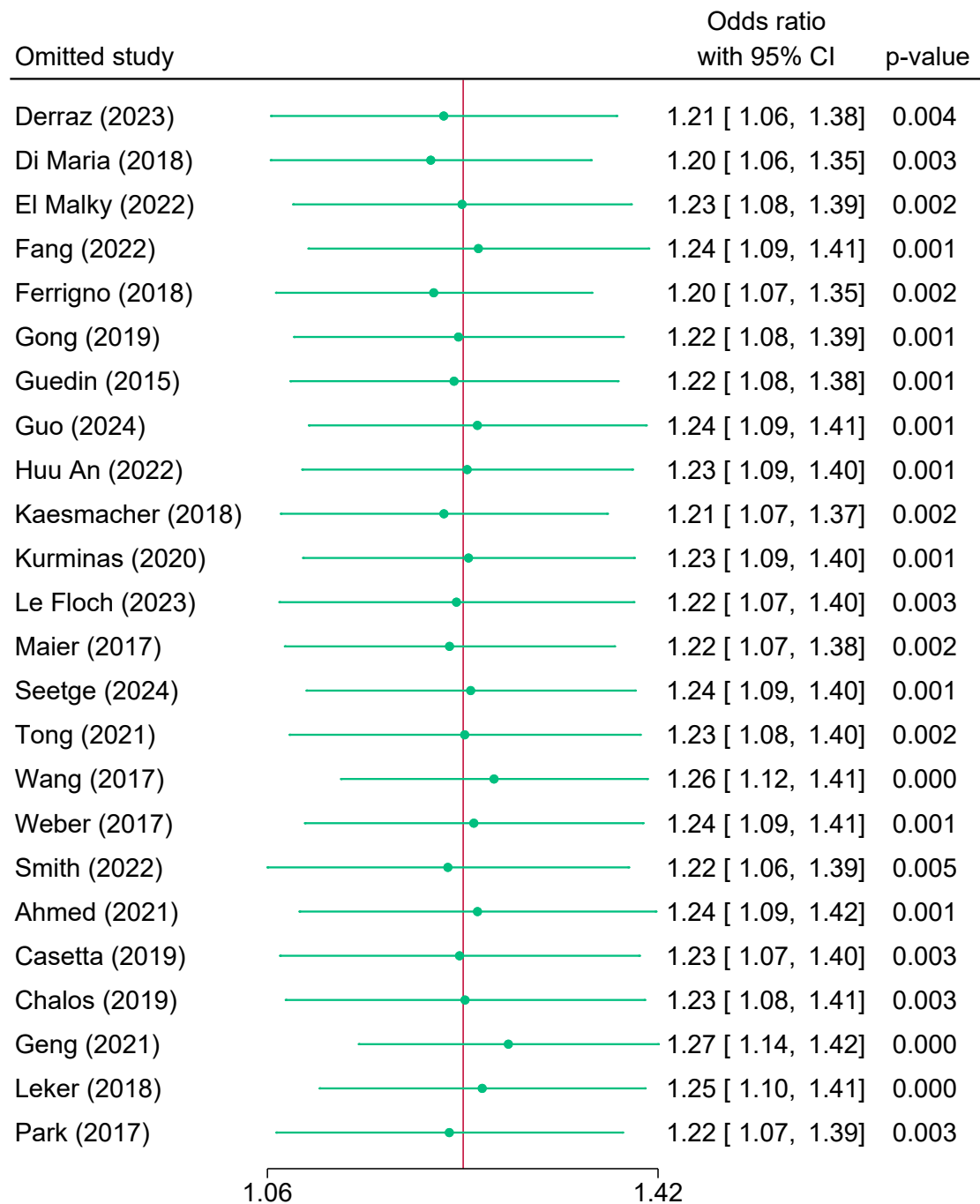

Random-effects REML model

Supplement: SUPPLEMENTARY FIGURE S5 — Sensitivity analysis of successful reperfusion outcome between bridging therapy and direct mechanical thrombectomy. [file Data_Sheet_5.PDF]

Log odds-ratio

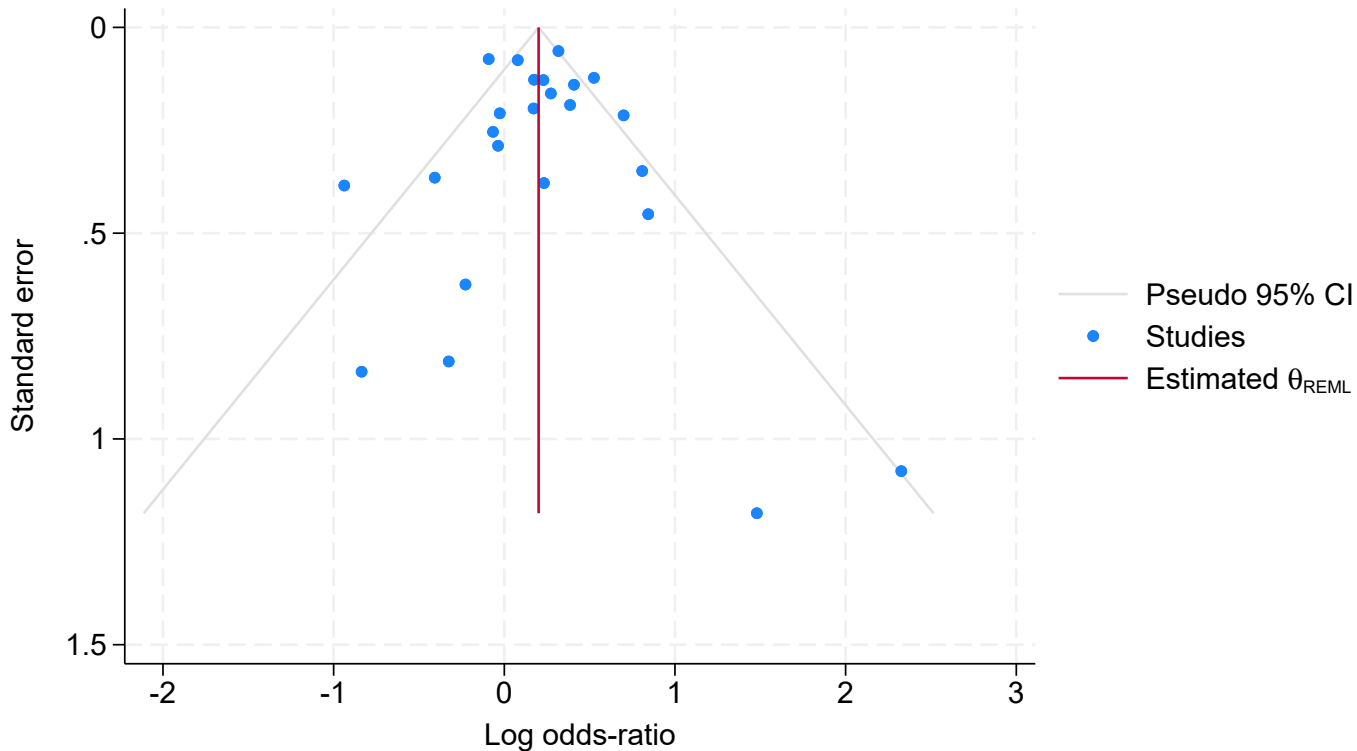

Supplement: SUPPLEMENTARY FIGURE S6 — Funnel plot showing the risk of publication bias in the successful reperfusion outcome. [file Data_Sheet_6.PDF]

# Funnel plot

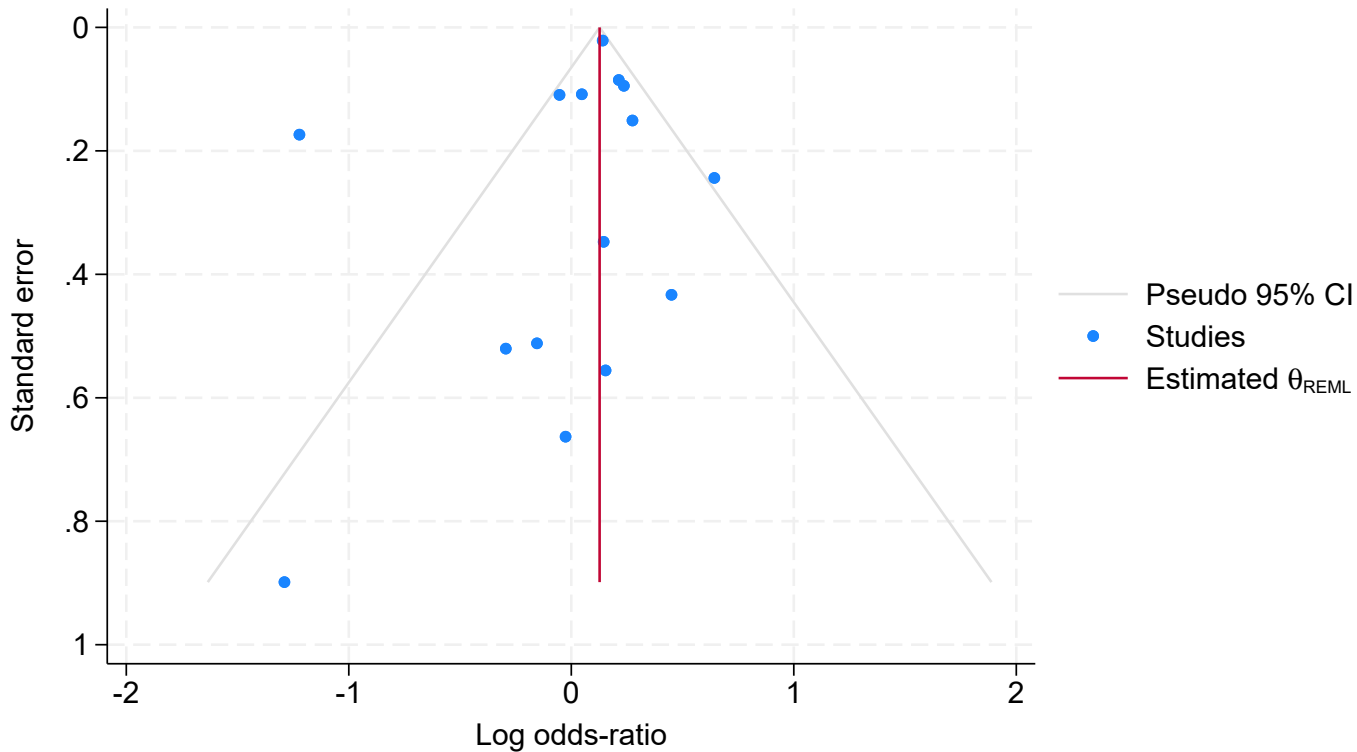

Supplement: SUPPLEMENTARY FIGURE S7 — Funnel plot showing the risk of publication bias in the any intracranial hemorrhage outcome. [file Data_Sheet_7.PDF]

# Funnel plot

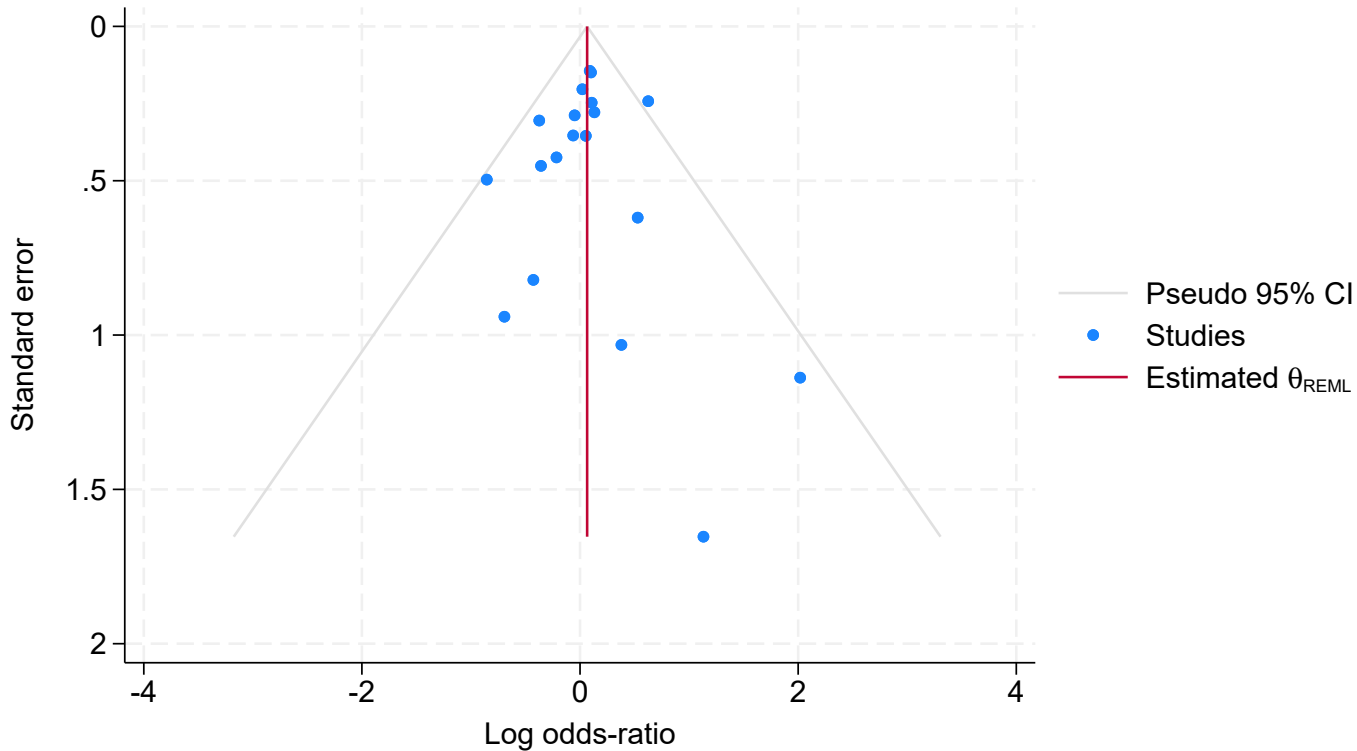

Supplement: SUPPLEMENTARY FIGURE S8 — Funnel plot showing the risk of publication bias in the symptomatic intracranial hemorrhage outcome. [file Data_Sheet_8.PDF]

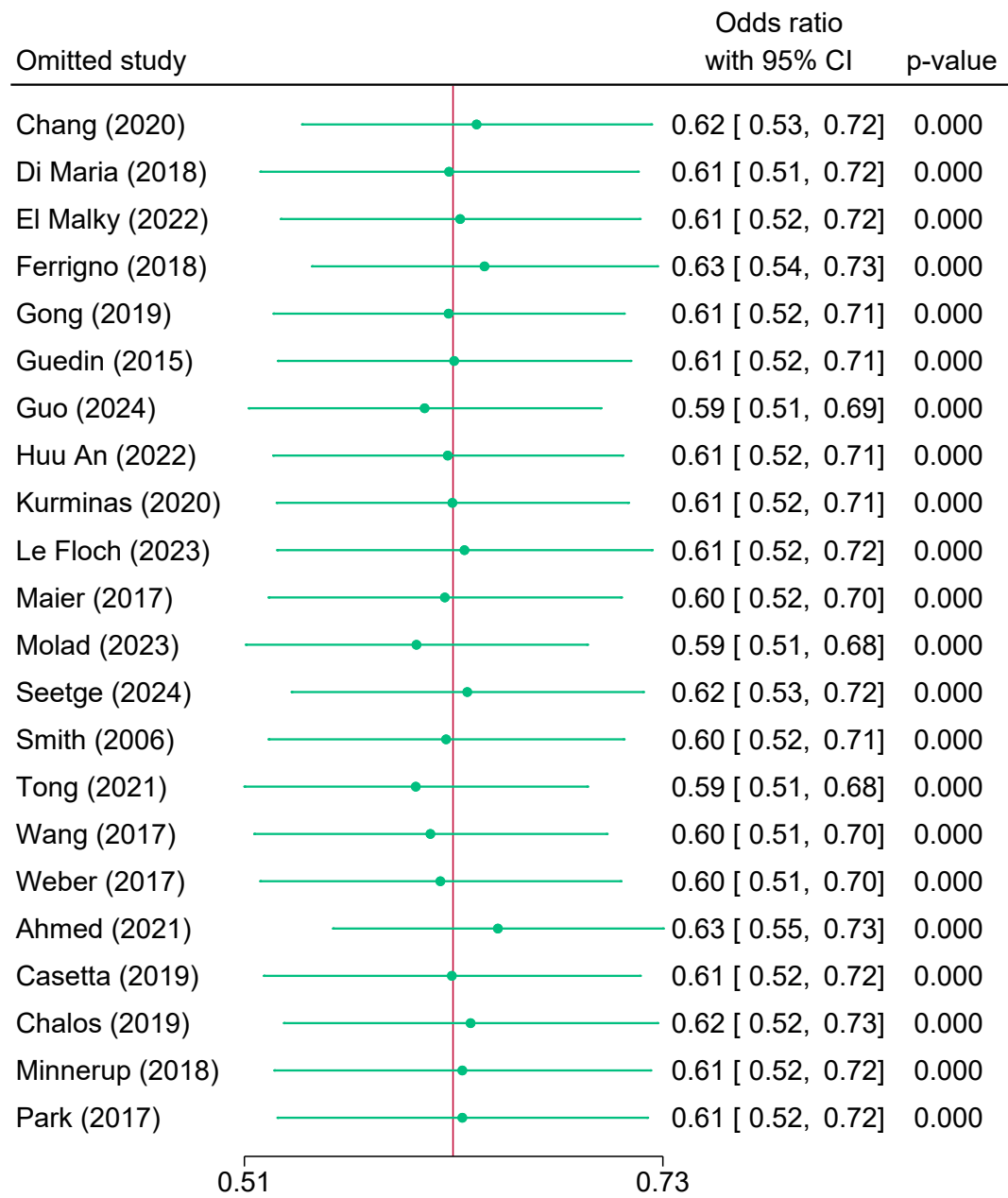

Random-effects REML model

Supplement: SUPPLEMENTARY FIGURE S9 — Sensitivity analysis of mortality risk between bridging therapy and direct mechanical thrombectomy. [file Data_Sheet_9.PDF]

# Funnel plot

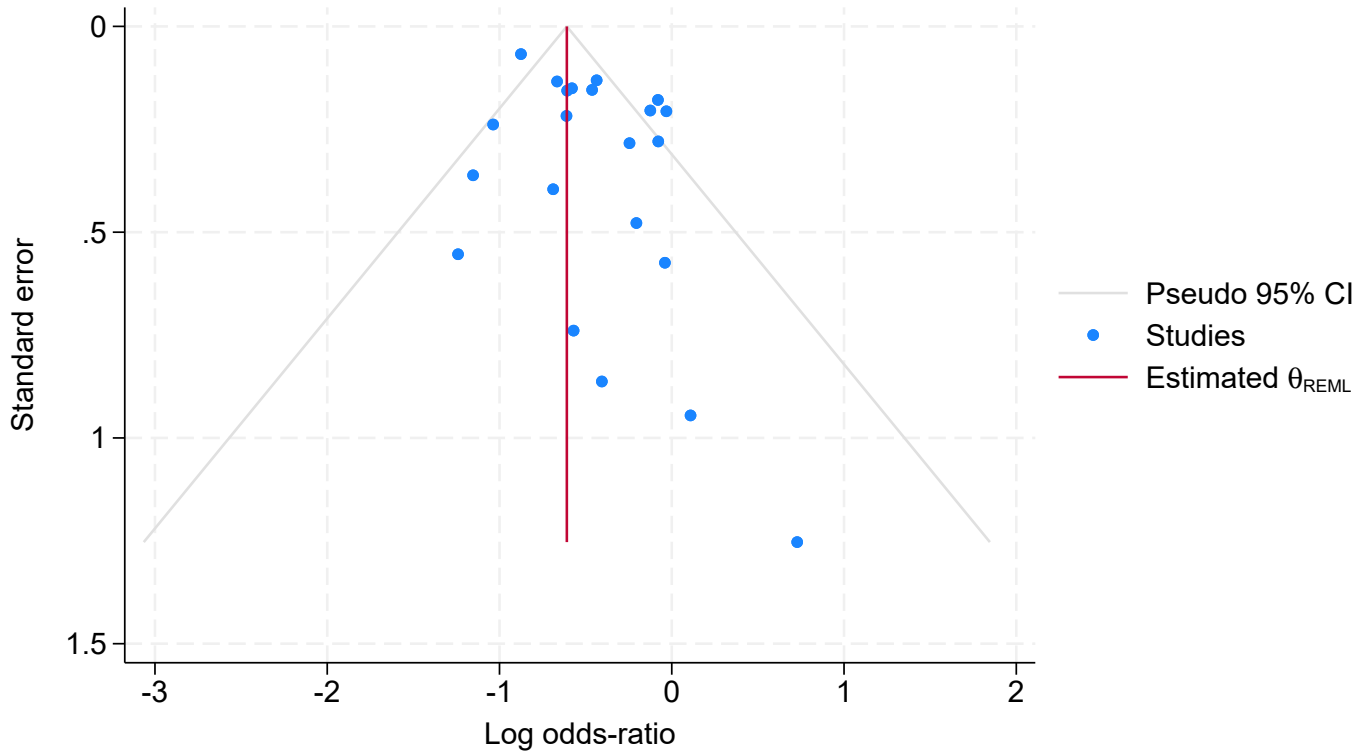

Supplement: SUPPLEMENTARY FIGURE S10 — Funnel plot showing the risk of publication bias in the mortality outcome. [file Data_Sheet_10.PDF]
