# Supplementary material for: Bridging therapy versus direct mechanical thrombectomy in acute ischemic stroke: an updated meta-analysis of real-world evidence
Source: Front Med (Lausanne). 2026 Jan 9;12:1731626. doi: 10.3389/fmed.2025.1731626 (PMC12827676; doi:10.3389/fmed.2025.1731626)
Supplement: Supplementary file 11 [file Supplementary_file_1.docx]

**Table S1.** The detailed search query employed in the database search

| No. | Search query | Results |
| --- | --- | --- |
| PubMed | | |
| #1 | thrombolysis[tiab] OR “thrombolytic therapy”[tiab] OR fibrinoly*[tiab] OR IVT[tiab] OR alteplase[tiab] OR tPA[tiab] OR "tissue plasminogen activator"[tiab] OR "bridging therapy"[tiab] OR "combined therapy"[tiab] OR activase[tiab] OR Actilyse[tiab] OR rtPA[tiab] OR Tenecteplase[tiab] OR TNKase[tiab] OR TNK-tPA[tiab] OR Reteplase[tiab] OR rPA[tiab] OR retavase[tiab] OR Desmoteplase[tiab] OR "Tissue Plasminogen Activator"[Mesh] | 136562 |
| #2 | Intravenous* | 507468 |
| #3 | “ischemic stroke”[tiab] OR “Cerebrovascular disease”[tiab] OR “Cerebrovascular accident”[tiab] OR “Brain ischemia”[tiab] OR “Brain Infarction”[tiab] OR “Thrombotic Stroke”[tiab] OR “cerebral infarction”[tiab] OR AIS[tiab] OR "Ischemic Stroke"[Mesh] | 144323 |
| #4 | Thrombectomy[tiab] OR “mechanical thrombolysis”[tiab] OR “endovascular procedure”[tiab] OR “clot retrieval”[tiab] OR “stent retriever”[tiab] OR “endovascular therapy”[tiab] OR “endovascular treatment”[tiab] OR "Thrombectomy"[Mesh] | 46341 |
| #5 | Random*[tiab] | 1629637 |
| #6 | Before[tiab] OR “pre-“[tiab] OR prior[tiab] OR preceding[tiab] OR preceded[tiab] | 1702322 |
| #7 | #1 AND #2 AND #3 AND #4 AND #5 AND #6 | 123 |
| Scopus | | |
| #1 | TITLE-ABS-KEY (thrombolysis) OR TITLE-ABS-KEY (“thrombolytic therapy”) OR TITLE-ABS-KEY (fibrinoly*) OR TITLE-ABS-KEY (IVT) OR TITLE-ABS-KEY (alteplase) OR TITLE-ABS-KEY (tPA) OR TITLE-ABS-KEY ("tissue plasminogen activator") OR TITLE-ABS-KEY ("bridging therapy") OR TITLE-ABS-KEY ("combined therapy") OR TITLE-ABS-KEY (activase) OR TITLE-ABS-KEY (Actilyse) OR TITLE-ABS-KEY (rtPA) OR TITLE-ABS-KEY (Tenecteplase) OR TITLE-ABS-KEY (TNKase) OR TITLE-ABS-KEY (TNK-tPA) OR TITLE-ABS-KEY (Reteplase) OR TITLE-ABS-KEY (rPA) OR TITLE-ABS-KEY (retavase) OR TITLE-ABS-KEY (Desmoteplase) | 242193 |
| #2 | ALL (Intravenous*) | 1642865 |
| #3 | TITLE-ABS-KEY (“ischemic stroke”) OR TITLE-ABS-KEY (“Cerebrovascular disease”) OR TITLE-ABS-KEY (“Cerebrovascular accident”) OR TITLE-ABS-KEY (“Brain ischemia”) OR TITLE-ABS-KEY (“Brain Infarction”) OR TITLE-ABS-KEY (“Thrombotic Stroke”) OR TITLE-ABS-KEY (“cerebral infarction”) OR TITLE-ABS-KEY (AIS) | 535003 |
| #4 | TITLE-ABS-KEY (Thrombectomy) OR TITLE-ABS-KEY (“mechanical thrombolysis”) OR TITLE-ABS-KEY (“endovascular procedure”) OR TITLE-ABS-KEY (“clot retrieval”) OR TITLE-ABS-KEY (“stent retriever”) OR TITLE-ABS-KEY (“endovascular therapy”) OR TITLE-ABS-KEY (“endovascular treatment”) | 85566 |
| #5 | TITLE-ABS-KEY (Random*) | 3568296 |
| #6 | TITLE-ABS-KEY (Before) OR TITLE-ABS-KEY (“pre-“) OR TITLE-ABS-KEY (prior) OR TITLE-ABS-KEY (preceding) OR TITLE-ABS-KEY (preceded) | 3586656 |
| #7 | #1 AND #2 AND #3 AND #4 AND #5 AND #6 | 256 |
| Web of Science | | |
| #1 | AB=thrombolysis OR AB=“thrombolytic therapy” OR AB=fibrinoly* OR AB=IVT OR AB=alteplase OR AB=tPA OR AB="tissue plasminogen activator" OR AB="bridging therapy" OR AB="combined therapy" OR AB=activase OR AB=Actilyse OR AB=rtPA OR AB=Tenecteplase OR AB=TNKase OR AB=TNK-tPA OR AB=Reteplase OR AB=rPA OR AB=retavase OR AB=Desmoteplase | 116880 |
| #2 | ALL=Intravenous* | 405970 |
| #3 | AB=“ischemic stroke” OR AB=“Cerebrovascular disease” OR AB=“Cerebrovascular accident” OR AB=“Brain ischemia” OR AB=“Brain Infarction” OR AB=“Thrombotic Stroke” OR AB=“cerebral infarction” OR AB=AIS | 122712 |
| #4 | AB=Thrombectomy OR AB=“mechanical thrombolysis” OR AB=“endovascular procedure” OR AB=“clot retrieval” OR AB=“stent retriever” OR AB=“endovascular therapy” OR AB=“endovascular treatment” | 32463 |
| #5 | AB=Random* | 2369198 |
| #6 | AB=Before OR AB=“pre-“ OR AB=prior OR AB=preceding OR AB=preceded | 4647342 |
| #7 | #1 AND #2 AND #3 AND #4 AND #5 AND #6 | 171 |
| Google Scholar | | |
| With all of the words | tissue plasminogen activator intravenous ischemic stroke thrombectomy random | - |
| With the exact phrase | - | - |
| With at least one of the words | Before prior pre- preceding preceded | - |
| Total | - | 200 |

Table S2. A complete list of excluded articles along with the reasons for exclusion

| ID | Author | Year of publication | Title | Reason for Exclusion |
| --- | --- | --- | --- | --- |
| 74 | Á. A. Chamorro, Sergio Castellanos, Mar Gomis, Meritxell Urra, Xabier Blasco, Jordi Arenillas, Juan F Román, Luis S Muñoz, Roberto Macho, Juan | 2017 | Uric acid therapy improves the outcomes of stroke patients treated with intravenous tissue plasminogen activator and mechanical thrombectomy | Use of IVT before ET not stated |
| 92 | S. B. A. Coutts, S. Appireddy, R. Arenillas, J. F. Assis, Z. Bailey, P. Barber, P. A. Bazan, R. Buck, B. H. Butcher, K. S. Camden, M. C. Campbell, B. C. V. Casaubon, L. K. Catanese, L. Chatterjee, K. Choi, P. M. C. Clarke, B. Dowlatshahi, D. Ferrari, J. Field, T. S. Ganesh, A. Ghia, D. Goyal, M. Greisenegger, S. Halse, O. Horn, M. Hunter, G. Imoukhuede, O. Kelly, P. J. Kennedy, J. Kenney, C. Kleinig, T. J. Krishnan, K. Lima, F. Mandzia, J. L. Marko, M. Martins, S. O. Medvedev, G. Menon, B. K. Mishra, S. M. Molina, C. Moussaddy, A. Muir, K. W. Parsons, M. W. Penn, A. M. W. Pille, A. Pontes-Neto, O. M. Roffe, C. Serena, J. Simister, R. Singh, N. Spratt, N. Strbian, D. Tham, C. H. Wiggam, M. I. Williams, D. J. Willmot, M. R. Wu, T. Yu, A. Y. X. Zachariah, G. Zafar, A. Zerna, C. Hill, M. D. Salluzzi, M. Blenkin, N. Dueck, A. Doram, C. Zhang, Q. Ryckborst, K. Bohn, S. Collier, Q. Taylor, F. Lethebe, B. C. Jambula, A. Sage, K. Toussaint, L. Save, S. Lee, J. Laham, N. Sultan, A. A. Deepak, A. Sitaram, A. Demchuk, A. M. Lockey, A. Micielli, A. Wadhwa, A. Arabambi, B. Graham, B. Bogiatzi, C. Doshi, D. Chakraborty, D. Kim, D. Vasquez, D. Singh, D. Tse, D. Harrison, E. Smith, E. E. Teleg, E. Klourfeld, E. Klein, G. Sebastian, I. A. Evans, J. Hegedus, J. Kromm, J. Lin, K. Ignacio, K. Ghavami, K. Ismail, M. Moores, M. Panzini, M. A. Boyko, M. Almekhlafi, M. A. Newcommon, N. Maraj, N. Volny, O. Stys, P. Couillard, P. Ojha, P. Eswaradass, P. Joundi, R. Singh, R. Asuncion, R. M. Muir, R. T. Dey, S. Mansoor, S. Wasyliw, S. Nagendra, S. Hu, S. Althubait, S. Chen, S. Bal, S. Van Gaal, S. Peters, S. Ray, S. Chaturvedi, S. Subramaniam, S. Fu, V. Villaluna, K. Maclean, G. King-Azote, P. Ma, C. Plecash, A. Murphy, C. Gorman, J. Wilson, L. Zhou, L. Benevente, O. Teal, P. Yip, S. Mann, S. Dewar, B. Demetroff, M. Shamloul, R. Beardshaw, R. Roberts, S. Blaquiere, D. Stotts, G. Shamy, M. Bereznyakova, O. Fahed, R. Alesefir, W. Lavoie, S. Hache, A. Collard, K. Mackey, A. Gosselin-Lefebvre, S. Verreault, S. Beauchamp, B. Lambourn, L. Khaw, A. Mai, L. Sposato, L. Bres Bullrich, M. Azarpazhooh, R. Fridman, S. Kapoor, A. Southwell, A. Bardi, E. Fatakdawala, I. Kamra, M. Lopes, K. Popel, N. Norouzi, V. Liu, A. Liddy, A. M. Ghoari, B. Hawkes, C. Enriquez, C. A. Gladstone, D. J. Manosalva Alzate, H. A. Khosravani, H. Hopyan, J. J. Sivakumar, K. Son, M. Boulos, M. I. Hamind, M. A. Swartz, R. H. Murphy, R. Reiter, S. Fitzpatrick, T. Bhandari, V. Good, J. Penn, M. Naylor, M. Frost, S. Cayley, A. Akthar, F. Williams, J. Kalman, L. Crellin, L. Wiegner, R. Singh, R. S. Stewart, T. To, W. Singh, S. Pikula, A. Jaigobin, C. Carpani, F. Silver, F. Janssen, H. Schaafsma, J. del Campo, M. Alskaini, M. Rajendram, P. Fairall, P. Granfield, B. Crawford, D. Jabs, J. White, L. Sivakumar, L. Piquette, L. Nguyen, T. Nomani, A. Wagner, A. Alrohimi, A. Butt, A. D'Souza, A. Gajurel, B. Vekhande, C. Kamble, H. Kalashyan, H. Lloret, M. Benguzzi, M. Arsalan, N. Ishaque, N. Ashayeriahmadabad, R. Samiento, R. Hosseini, S. Kazi, S. Das, S. Sugumar, T. Selchen, D. Kostyrko, P. Muccilli, A. Saposnik, A. G. Vandervelde, C. Ratnayake, K. McMillan, S. Katsanos, A. Shoamanesh, A. Sahlas, D. J. Naidoo, V. Todorov, V. Toma, H. Brar, J. Lee, J. Horton, M. Shand, E. Weatherby, S. Jin, A. Durafourt, B. Jalini, S. Gardner, A. Tyson, C. Junk, E. Foster, K. Bolt, K. Sylvain, N. Maley, S. Urroz, L. Peeling, L. Kelly, M. Whelan, R. Cooley, R. Teitelbaum, J. Boutayeb, A. Moore, A. Cole, E. Waxman, L. Ben-Amor, N. Sanchez, R. Khalil, S. Nehme, A. Legault, C. Tampieri, D. Ehrensperger, E. Vieira, L. Cortes, M. Angle, M. Hannouche, M. Badawy, M. Werner, K. Wieszmuellner, S. Langer, A. Gisold, A. Zach, H. Rommer, P. Macher, S. Blechinger, S. Marik, W. Series, W. Baumgartinger, M. Krebs, S. Koski, J. Eirola, S. Ivanoff, T. Erakanto, A. Kupari, L. Sibolt, G. Panula, J. Tomppo, L. Tiainen, M. Ahlstrom, M. Martinez Majander, N. Suomalainen, O. Raty, S. Levi, C. Kerr, E. Allen, J. Kaauwai, L. P. Belevski, L. Russell, M. Ormond, S. Chew, A. Loiselle, A. Royan, A. Hughes, B. Garcia Esperon, C. Pepper, E. Miteff, F. He, J. Lycett, M. Min, M. Murray, N. Pavey, N. Starling de Barros, R. Gangadharan, S. Dunkerton, S. Waller, S. Canento Sanchez, T. Wellings, T. Edmonds, G. Whittaker, K. A. Ewing, M. Lee, P. Singkang, R. McDonald, A. Dos Santos, A. Shin, C. Jackson, D. Tsoleridis, J. Fisicchia, L. Parsons, N. Shenoy, N. Smith, S. Sharobeam, A. Balabanski, A. Park, A. Williams, C. Pavlin-Premri, D. Rodrigues, E. Alemseged, F. Ng, F. Zhao, H. Beharry, J. Ng, J. L. Williamson, J. Wong, J. Z. W. Li, K. Kwan, M. K. Valente, M. Yassi, N. Yogendrakumar, V. McNamara, B. Buchanan, C. McCarthy, C. Thomas, G. Stephens, K. Chung, M. Chung, M. F. Tang, M. Busch, T. Frost, T. Lee, R. Stuart, N. Pachani, N. Menon, A. Borojevic, B. Linton, C. M. Garcia, G. Callaly, E. P. Dewey, H. Liu, J. Chen, J. Wong, J. Nowak, K. To, K. Lizak, N. S. Bhalala, O. Park, P. Tan, P. Martins, R. Cody, R. Forbes, R. Chen, S. K. Ooi, S. Tu, S. Dang, Y. L. Ling, Z. Cranefield, J. Drew, R. Tan, A. Kurunawai, C. Harvey, J. Mahadevan, J. J. Cagi, L. Palanikumar, L. Chia, L. N. Goh, R. El-Masri, S. Urbi, B. Rapier, C. Berrill, H. McEvoy, H. Dunning, R. Kuriakose, S. Chad, T. Sapaen, V. Sabet, A. Shah, D. Yeow, D. Lilley, K. Ward, K. Mozhy Mahizhnan, M. Tan, M. Lynch, C. Coveney, S. Tobin, K. McCabe, J. Marnane, M. Murphy, S. Large, M. Moynihan, B. Boyle, K. Sanjuan, E. Sanchis, M. Boned, S. Pancorbo, O. Sala, V. Garcia, L. Garcia-Tornel, A. Juega, J. Pagola, J. Santana, K. Requena, M. Muchada, M. Olive, M. Lozano, P. J. Rubiera, M. Deck, M. Rodriguez, N. Gomez, B. Reyes Munoz, F. J. Gomez, A. S. Sanz, A. C. Garcia, E. C. Penacoba, G. Ramos, M. E. de Lera Alfonso, M. Feliu, A. Pardo, L. Ramirez, P. Murillo, A. Lopez Dominguez, D. Rodriguez, J. Terceno Izaga, M. Reina, M. Viturro, S. B. Bojaryn, U. Vera Monge, V. A. Silva Blas, Y. R Siew, R. Agustin, S. J. Seet, C. Tianming, T. d'Emden, A. Murray, A. Welch, A. Hatherley, K. Day, N. Smith, W. MacRae, E. Mitchell, E. S. Mahmood, A. Elliot, J. Neilson, S. Biswas, V. Brown, C. Lewis, A. Ashton, A. Werring, D. Perry, R. Muhammad, R. Lee, Y. C. Black, A. Robinson, A. Williams, A. Banaras, A. Cahoy, C. Raingold, G. Marinescu, M. Atang, N. Bason, N. Francia, N. Obarey, S. Feerick, S. Joseph, J. Schulz, U. Irons, R. Benjamin, J. Quinn, L. Jhoots, M. Teal, R. Ford, G. Harston, G. Bains, H. Gbinigie, I. Mathieson, P. Sim, C. H. Hayter, E. Kennedy, K. Binnie, L. Priestley, N. Williams, R. Ghatala, R. Stratton, S. Blight, A. Zhang, L. Davies, A. Duffy, H. Roberts, J. Homer, J. Roberts, K. Dodd, K. Cawley, K. Martin, M. Leason, S. Cotgreave, S. Taylor, T. Nallasivan, A. Haider, S. Chakraborty, T. Webster, T. Gil, A. Martin, B. Joseph, B. Cabrera, C. Jose, D. Man, J. Aquino, J. Sebastian, S. Osterdahl, M. Matthew, M. Ike, N. Bello, P. Wilding, P. Fuentes, R. Shah, R. Mashate, S. Patel, T. Nwanguma, U. Dave, V. Haber, A. Lee, A. O'Sullivan, A. Drumm, B. Dawson, A. C. Matar, T. Roberts, D. Taylor, E. Rounis, E. El-Masry, A. O'Hare, C. Kalladka, D. Jamil, S. Auger, S. Raha, O. Evans, M. Vonberg, F. Kalam, S. Ali Sheikh, A. Jenkins, I. H. George, J. Kwan, J. Blagojevic, J. Saeed, M. Haji-Coll, M. Tsuda, M. Sayed, M. Winterkron, N. Thanbirajah, N. Vittay, O. Karim, R. Smail, R. C. Gauhar, S. Elmamoun, S. Malani, S. Pralhad Kelavkar, S. Hiden, J. Ferdinand, P. Sanyal, R. Varquez, R. Smith, B. Okechukwu, C. Fox, E. Collins, E. Courtney, K. Tauro, S. Patterson, C. McShane, D. Roberts, G. McIimoyle, J. McGuire, K. Fearon, P. Gordon, P. Isaacs, K. Lucas, K. Smith, L. Dews, L. Bates, M. Lawrence, S. Heeley, S. Patel, V. Chin, Y. M. Sims, D. Littleton, E. Khaira, J. Nadar, K. Kieliszkowska, A. Sari, B. Domingos Belo, C. Smith, E. Manolo, E. Y. Aeron-Thomas, J. Doheny, M. Garcia Pardo, M. Recaman, M. Tibajia, M. C. Aissa, M. Mah, Y. Yu, T. Meenakshisundaram, S. Heller, S. Alsukhni, R. Williams, O. Farag, M. Benger, M. Engineer, A. Bayhonan, S. Conway, S. Bhalla, A. Nouvakis, D. Theochari, E. Boyle, F. Teo, J. King-Robson, J. Law, K. Y. Sztriha, L. McGovern, A. Day, D. Mitchell-Douglas, J. Francis, J. Iqbal, A. Punjabivaryani, P. Anonuevo Reyes, J. Anonuevo Reyes, M. Pauls, M. Buch, A. Hedstrom, A. Hutchinson, C. Kirkland, C. Newham, J. Wilkes, G. Fleming, L. Fleck, N. Franca, A. Chwal, B. Oldoni, C. Mantovani, G. Noll, G. Zanella, L. Soma, M. Secchi, T. Borelli, W. Rimoli, B. P. da Cunha Silva, G. H. Machado Galvao Mondin, L. A. Barbosa Cerantola, R. Imthon, A. K. Esaki, A. S. Camilo, M. Vincenzi, O. C. ds Cruz, R. R. Morillos, M. B. Riccioppa Rodrigues, G. G. Santos Ferreira, K. Pazini, A. M. Pena Pereira, M. A. de Albuquerque, A. L. A. Massote Fontanini, C. E. Matinez Rubio, C. F. dos Santos, D. T. Dias, F. A. Alves, F. F. A. Milani, C. Pegorer Santos, B. Winckler, F. De Souza, J. T. Bonome, L. A. M. Cury Silva, V. A. Teodoro, R. S. Modolo, G. P. Ferreira, N. C. Barbosa dos Santos, D. F. dos Santos Moreira, J. C. Cruz Guedes de Morais, A. B. Vieira, J. Mendes, G. de Queiroz, J. P. | 2024 | Tenecteplase versus standard of care for minor ischaemic stroke with proven occlusion (TEMPO-2): a randomised, open label, phase 3 superiority trial | Use of IVT before ET not stated |
| 219 | A. R. Kastrup, C. Politi, M. Alexandrou, M. Hildebrandt, H. Schröter, A. Papanagiotou, P. | 2021 | Endovascular Therapy vs. Thrombolysis in Pre-stroke Dependent Patients With Large Vessel Occlusions Within the Anterior Circulation | Use of IVT before ET not stated |
| 423 | D. K. Tanne, Scott E Demchuk, Andrew M Koren-Morag, Nira Hanson, Sandra Grond, Martin Levine, Steven R Multicenter rt-PA Stroke Survey Group | 2002 | Markers of increased risk of intracerebral hemorrhage after intravenous recombinant tissue plasminogen activator therapy for acute ischemic stroke in clinical practice: the multicenter rt-PA acute stroke survey | Use of IVT before ET not stated |
| 485 | S. K. Yamamoto, Hiroshi Sutani, Yasuo Akita, Yuzo Otani, Hajime Iwasaka, Toshiji | 2006 | Effects of intravenous administration of tissue plasminogen activator before thrombectomy in patients with acute myocardial infarction | Use of IVT before ET not stated |
| 32 | J. S. S. Balami, B. A. Buchan, A. M. | 2013 | Complications associated with recombinant tissue plasminogen activator therapy for acute Ischaemic stroke | Use of IVT before ET not stated |
| 52 | G. M. Broocks, R. Bechstein, M. Hanning, U. Brekenfeld, C. Flottmann, F. Kniep, H. Nawka, M. T. Deb-Chatterji, M. Thomalla, G. Sporns, P. Yeo, L. L. L. Tan, B. Y. Q. Gopinathan, A. Kastrup, A. Politi, M. Papanagiotou, P. Kemmling, A. Fiehler, J. Meyer, L. German Stroke Registry Endovasc, Tr | 2023 | Benefit and risk of intravenous alteplase in patients with acute large vessel occlusion stroke and low ASPECTS | Use of IVT before ET not stated |
| 120 | Y. C. Dong, Wenjie Cheng, Xin Fang, Kun Wu, Fei Yang, Lumeng Xie, Yanan Dong, Qiang | 2016 | Low-dose intravenous tissue plasminogen activator for acute ischaemic stroke: an alternative or a new standard? | Use of IVT before ET not stated |
| 145 | G. C. S. Fonarow, Eric E Saver, Jeffrey L Reeves, Mathew J Bhatt, Deepak L Grau-Sepulveda, Maria V Olson, DaiWai M Hernandez, Adrian F Peterson, Eric D Schwamm, Lee H | 2011 | Timeliness of tissue-type plasminogen activator therapy in acute ischemic stroke: patient characteristics, hospital factors, and outcomes associated with door-to-needle times within 60 minutes | Use of IVT before ET not stated |
| 184 | J.-H. S. Hong, Sung-Il Kang, Jihoon Jang, Min Uk Kim, Beom Joon Han, Moon-Ku Park, Tai Hwan Park, Sang-Soon Lee, Kyung Bok Lee, Byung-Chul | 2016 | Endovascular treatment in patients with persistent internal carotid artery occlusion after intravenous tissue plasminogen activator: a clinical effectiveness study | Use of IVT before ET not stated |
| 195 | G. B. Imbarrato, Joshua Gordhan, Ajeet | 2018 | Clinical outcomes of endovascular thrombectomy in tissue plasminogen activator versus non-tissue plasminogen activator patients at primary stroke care centers | Use of IVT before ET not stated |
| 239 | E. S. S. Kristoffersen, D. J. Meinel, T. R. | 2025 | Intravenous thrombolysis and mechanical thrombectomy in acute stroke patients on direct oral anticoagulants | Use of IVT before ET not stated |
| 250 | M.-H. I. Lee, Sang-Hyuk Jo, Kwang Wook Yoo, Do-Sung | 2023 | Recanalization Rate and Clinical Outcomes of Intravenous Tissue Plasminogen Activator Administration for Large Vessel Occlusion Stroke Patients | Use of IVT before ET not stated |
| 251 | R. R. E. Leker, Roni Gomori, John M de Noriega, Fernando Ramirez Ben-Hur, Tamir Cohen, Jose E | 2012 | Stent-based thrombectomy versus intravenous tissue plasminogen activator in patients with acute middle cerebral artery occlusion | Use of IVT before ET not stated |
| 267 | Y. L. Luo, Jinming Huang, Ligang Liu, Xiaochuan Zhang, Boyu Lin, Jixian Jiang, Aihua Zhao, Jing | 2022 | Safety and efficacy of a new modified intravenous recombinant tissue plasminogen activator (rt-PA) regimen in Chinese patients with acute ischemic stroke: A descriptive retrospective cohort study with subgroup-analysis of different rt-PA dose | Use of IVT before ET not stated |
| 275 | P. P. Malik, Urvish K Kaul, Surabhi Singla, Ramit Kavi, Tapan Arumaithurai, Kogulavadanan Jani, Vishal B | 2021 | Risk factors and outcomes of intravenous tissue plasminogen activator and endovascular thrombectomy utilization amongst pediatrics acute ischemic stroke | Use of IVT before ET not stated |
| 276 | S. S. Man, Nicole Mac Grory, Brian Alhanti, Brooke Uchino, Ken Saver, Jeffrey L Smith, Eric E Xian, Ying Bhatt, Deepak L Schwamm, Lee H | 2023 | Shorter door-to-needle times are associated with better outcomes after intravenous thrombolytic therapy and endovascular thrombectomy for acute ischemic stroke | Use of IVT before ET not stated |
| 300 | J. W. Minnerup, Heike Teuber, Anja Wellmann, Jürgen Eyding, Jens Weber, Ralph Reimann, Gernot Weber, Werner Krause, Lars Udo Kurth, Tobias | 2016 | Outcome after thrombectomy and intravenous thrombolysis in patients with acute ischemic stroke: a prospective observational study | Use of IVT before ET not stated |
| 325 | Y. C. M. Ooi, Brian Behdad Mukarram, Faisal Kaneko, Naoki Nour, May Colby, Geoffrey Jahan, Reza Tateshima, Satoshi Duckwiler, Gary Saver, Jeffrey | 2021 | Role of Intravenous Tissue Plasminogen Activator in Acute Ischemic Stroke with Large Vessel Occlusion | Use of IVT before ET not stated |
| 327 | O. O. Ozdemir, Z. Vural, M. Durmaz, R. Cosan, E. Arslantas, A. Atasoy, M. A. | 2014 | Early decompressive surgery after combined intra-venous thrombolysis and endovascular stroke treatment | Use of IVT before ET not stated |
| 342 | Z. L. Qiu, Fengli Xie, Dongjing Yuan, Guangxiong Nguyen, Thanh N Zhou, Kai Nogueira, Raul G Saver, Jeffrey L Campbell, Bruce CV Albers, Gregory W | 2024 | Efficacy and Safety of Intravenous Tenecteplase Before Endovascular Thrombectomy for Acute Ischemic Stroke: The Multicenter, Randomized, BRIDGE‐TNK Trial Protocol | Use of IVT before ET not stated |
| 371 | R. N. Saha, Gaurav Solanki, Dhanshree Shaheen, Ahmed Al-Salihi, Mohammed Maan Dalal, Shamser Singh Roy, Anil | 2025 | Endovascular thrombectomy versus intravenous tissue plasminogen activator for vertebrobasilar stroke treatment: insights from the national inpatient sample | Use of IVT before ET not stated |
| 372 | T. R. Sairanen, J. | 2019 | Should we thrombolyse prior to endovascular treatment in acute stroke? | Use of IVT before ET not stated |
| 373 | H. A. Y. Salim, V. Musmar, B. Adeeb, N. Essibayi, M. A. El Naamani, K. Henninger, N. Sundararajan, S. H. Kuehn, A. L. Khalife, J. Ghozy, S. Scarcia, L. Tan, B. Y. Q. Pulli, B. Heit, J. J. Regenhardt, R. W. Cancelliere, N. M. Bernstock, J. D. Rouchaud, A. Fiehler, J. Sheth, S. Puri, A. S. Dyzmann, C. Colasurdo, M. Barreau, X. Renieri, L. Filipe, J. P. Harker, P. Radu, R. A. Marotta, T. R. Spears, J. Ota, T. Mowla, A. Jabbour, P. Biswas, A. Clarencon, F. Siegler, J. E. Nguyen, T. N. Varela, R. Baker, A. Altschul, D. Gonzalez, N. R. Moehlenbruch, M. A. Costalat, V. Gory, B. Stracke, C. P. Aziz-Sultan, M. A. Hecker, C. Shaikh, H. Liebeskind, D. S. Pedicelli, A. Alexandre, A. M. Tancredi, I. Faizy, T. D. Kalsoum, E. Lubicz, B. Patel, A. B. Pereira, V. M. Guenego, A. Dmytriw, A. A. Mad Mt Investigators | 2024 | Mechanical Thrombectomy Versus Intravenous Thrombolysis in Distal Medium Vessel Acute Ischemic Stroke: A Multinational Multicenter Propensity Score-Matched Study | Use of IVT before ET not stated |
| 409 | E. E. Z. Smith, C. Solomon, N. Matsouaka, R. Mac Grory, B. Saver, J. L. Hill, M. D. Fonarow, G. C. Schwamm, L. H. Messé, S. R. Xian, Y. | 2022 | Outcomes After Endovascular Thrombectomy With or Without Alteplase in Routine Clinical Practice | Use of IVT before ET not stated |
| 435 | C. J. Traenka, S. Gralla, J. Kurmann, R. Stippich, C. Simonetti, B. G. Gensicke, H. Mueller, H. Lovblad, K. Eskandari, A. Puccinelli, F. Vehoff, J. Weber, J. Wegener, S. Steiner, L. Kägi, G. Luft, A. Sztajzel, R. Fischer, U. Bonati, L. H. Peters, N. Michel, P. Lyrer, P. A. Arnold, M. Engelter, S. T. | 2018 | Endovascular therapy versus intravenous thrombolysis in cervical artery dissection ischemic stroke - Results from the SWISS registry | Use of IVT before ET not stated |
| 496 | T. U. Yoshie, T. Hasegawa, Y. Takeuchi, M. Morimoto, M. Tsuboi, Y. Yamamoto, R. Kaku, S. Ayabe, J. Akiyama, T. Yamamoto, D. Mori, K. Kagami, H. Ito, H. Onodera, H. Kaga, Y. Ohtsubo, H. Tatsuno, K. Usuki, N. Takaishi, S. Yamano, Y. K. Net Registry Investigators | 2024 | Endovascular Thrombectomy Versus Intravenous Alteplase For Distal Medium Vessel Occlusions: A Propensity Score-Matched Analysis | Use of IVT before ET not stated |
| 29 | X. Q. Bai, J. Wang, Y. | 2023 | Endovascular thrombectomy with or without intravenous alteplase in acute stroke: a systematic review and meta-analysis of randomized clinical trials | REVIEW |
| 55 | L. Y. W. Cai, L. Y. Campbell, B. C. V. Wu, Y. L. Abdalkader, M. Alemseged, F. Kaesmacher, J. Puetz, V. Nagel, S. Strbian, D. Knapen, Rrmm Li, C. H. Ye, S. T. Tian, P. L. Chen, J. J. Li, R. T. Hu, W. Qiu, Z. M. Nguyen, T. N. Schonewille, W. J. Guo, Q. F. Dai, Z. | 2024 | Endovascular thrombectomy with versus without intravenous thrombolysis in patients with acute basilar artery occlusion: a systematic review and meta-analysis | REVIEW |
| 79 | J. W. Chen, Teng-Fei Xu, Tian-Ce Chang, Guo-Can Chen, Hui-Sheng Liu, Liang | 2021 | Direct endovascular thrombectomy or with prior intravenous thrombolysis for acute ischemic stroke: a meta-analysis | REVIEW |
| 121 | H. L. Du, Hanhan Ambler, Gareth Fang, Shuangfang He, Raoli Yuan, Qilin Werring, David J Liu, Nan | 2021 | Intravenous thrombolysis before mechanical thrombectomy for acute ischemic stroke: a meta‐analysis | REVIEW |
| 123 | H. W. L. Du, H. H. Ambler, G. Fang, S. F. He, R. L. Yuan, Q. L. Werring, D. J. Liu, N. | 2021 | Intravenous Thrombolysis Before Mechanical Thrombectomy for Acute Ischemic Stroke: A Meta-Analysis | REVIEW |
| 134 | L. Z. Fan, Lin Liu, Xiaodong Wang, Jian Qiu, Jianting Wang, Yujie | 2021 | Outcomes of mechanical thrombectomy with pre-intravenous thrombolysis: a systematic review and meta-analysis | REVIEW |
| 155 | H. S. E. Ghaith, Mohamed Gabra, Mohamed Diaa Nawar, Asmaa Ahmed Abd-Alkhaleq, Mohamed Sameh Hamam, Khaled M Aboelnasr, Lara Ebrahim Elgezery, Esraa Ayman Osman, Mohamed Hosny Elsayed, Hanaa | 2022 | Intravenous thrombolysis before mechanical thrombectomy for acute ischemic stroke due to large vessel occlusion; should we cross that bridge? A systematic review and meta-analysis of 36,123 patients | REVIEW |
| 160 | M. C. Gottlieb, J. N. Westrick, J. Peksa, G. D. | 2025 | Endovascular thrombectomy with versus without intravenous thrombolysis for acute ischaemic stroke | REVIEW |
| 169 | S. Q. Guo, Shiran Tan, Sitao Su, Henghai Chen, Xiaoyu | 2024 | Endovascular thrombectomy without versus with different pre-intravenous thrombolysis in acute ischemic stroke: a network meta-analysis of randomized controlled trials | REVIEW |
| 173 | A. A.-Q. Hammed, A. Alzawahreh, A. Rosenbauer, J. Nada, E. A. Otmani, Z. Hamam, N. G. Alnajjar, A. Z. Hammad, E. M. Hamamreh, R. Kostev, K. Richter, G. Tanislav, C. | 2024 | Comparative Effectiveness of Intravenous Thrombolysis plus Mechanical Thrombectomy versus Mechanical Thrombectomy Alone in Acute Ischemic Stroke: A Systematic Review and Meta-Analysis | REVIEW |
| 185 | L. C. B. Horvath, Felix Hosmann, Arthur Greisenegger, Stefan Kammerer, Kerstin Jilma, Bernd Siller-Matula, Jolanta M Zeitlinger, Markus Gelbenegger, Georg Jorda, Anselm | 2023 | Endovascular thrombectomy with or without intravenous thrombolysis in large-vessel ischemic stroke: A non-inferiority meta-analysis of 6 randomised controlled trials | REVIEW |
| 198 | M. F. G. Ishfaq, S. Huang, W. Lobanova, I. Martin, R. H. French, B. R. Siddiq, F. Gurkas, E. Aytac, E. Gomez, C. R. Qureshi, A. I. | 2023 | Endovascular Thrombectomy With or Without Intravenous Thrombolysis: A Meta-Analysis of Randomized Controlled Trials | REVIEW |
| 223 | A. H. T. Katsanos, G. Psychogios, M. Kaesmacher, J. Palaiodimou, L. Stefanou, M. I. Magoufis, G. Shoamanesh, A. Themistocleous, M. Sacco, S. Fiehler, J. Gralla, J. Strbian, D. Alexandrov, A. V. Fischer, U. Tsivgoulis, G. | 2021 | Utility of Intravenous Alteplase Prior to Endovascular Stroke Treatment: A Systematic Review and Meta-analysis of RCTs | REVIEW |
| 254 | H. Y. Li, Siyuan Zhong, Yi Wang, Jiahe Li, Xiang Gao, Heng Chen, Gang | 2022 | Mechanical thrombectomy with or without intravenous thrombolysis in acute ischemic stroke: a meta-analysis for randomized controlled trials | REVIEW |
| 258 | C.-H. S. Lin, Jeffrey L Ovbiagele, Bruce Huang, Wen-Yi Lee, Meng | 2022 | Endovascular thrombectomy without versus with intravenous thrombolysis in acute ischemic stroke: a non-inferiority meta-analysis of randomized clinical trials | REVIEW |
| 302 | E. A. M. Mistry, Akshitkumar M Nakawah, Mohammad Obadah Chitale, Rohan V James, Robert F Volpi, John J Fusco, Matthew R | 2017 | Mechanical thrombectomy outcomes with and without intravenous thrombolysis in stroke patients: a meta-analysis | REVIEW |
| 333 | A. D. Podlasek, Permesh Singh Butt, Waleed Grunwald, Iris Q England, Timothy J | 2021 | Direct mechanical thrombectomy without intravenous thrombolysis versus bridging therapy for acute ischemic stroke: a meta-analysis of randomized controlled trials | REVIEW |
| 454 | S. R. Vidale, Michele Consoli, Domenico Agostoni, Elio Clemente | 2020 | Bridging versus direct mechanical thrombectomy in acute ischemic stroke: a subgroup pooled meta-analysis for time of intervention, eligibility, and study design | REVIEW |
| 456 | S. R. Vidale, M. Clemente Agostoni, E. | 2021 | Mechanical thrombectomy with or without thrombolysis: A meta-analysis of RCTs | REVIEW |
| 465 | X. Y. Wang, Zhikang Busse, Jason W Hill, Michael D Smith, Eric E Guyatt, Gordon H Prasad, Kameshwar Lindsay, M Patrice Yang, Hui Zhang, Yi | 2022 | Endovascular thrombectomy with or without intravenous alteplase for acute ischemic stroke due to large vessel occlusion: a systematic review and meta-analysis of randomized trials | REVIEW |
| 466 | Z. J. Wang, Kangxiang Fang, Qi | 2024 | Endovascular thrombectomy with or without intravenous alteplase in large-core ischemic stroke: a systematic review and meta-analysis | REVIEW |
| 478 | X. G. Wu, Yi Chen, Shujun Yan, Zeya Wang, Zilan Zhang, Wei Chen, Zhouqing Xue, Tao Wang, Zhong | 2022 | Thrombectomy with or without thrombolysis in patients with acute ischemic stroke: a systematic review and meta-analysis | REVIEW |
| 504 | J. Y. Zhang, C. Deng, X. Yuan, Q. Wang, M. Fu, P. Fang, J. Du, Z. Hu, J. | 2022 | Efficacy and safety of endovascular treatment with or without intravenous alteplase in acute anterior circulation large vessel occlusion stroke: a meta-analysis of randomized controlled trials | REVIEW |
| 510 | W. L. Zheng, H. Lin, X. Liu, N. Tang, Y. Wu, J. Fang, S. Lin, Z. Xia, P. Du, H. | 2022 | Intravenous thrombolysis prior to endovascular treatment for acute ischemic stroke: a meta-analysis | REVIEW |
| 449 | S. A. U. V. van den Berg, S. M. LeCouffe, N. E. Postma, A. A. Lycklama À Nijeholt, G. J. Rinkel, L. A. Treurniet, K. M. Kappelhof, M. Bruggeman, A. E. van Kranendonk, K. R. Majoie, C. B. Dippel, D. W. van der Worp, H. B. Coutinho, J. M. Nederkoorn, P. J. Roos, Y. B. | 2023 | Admission blood pressure and clinical outcomes in patients with acute ischaemic stroke treated with intravenous alteplase and endovascular treatment versus endovascular treatment alone: A MR CLEAN-NO IV substudy | RCT |
| 512 | Y. W. Zhou, Zijun Ospel, Johanna Goyal, Mayank McDonough, Rosalie Yang, Pengfei Zhang, Yongwei Zhang, Lei Ye, Xiaofei Wei, Fulai | 2022 | Effect of admission hyperglycemia on safety and efficacy of intravenous alteplase before thrombectomy in ischemic stroke: post-hoc analysis of the DIRECT-MT trial | RCT |
| 513 | Y. X. Zhou, Pengfei Li, Zifu Zhang, Xiaoxi Zhang, Lei Zhang, Yongxin Zhang, Yongwei Hong, Bo Xu, Yi Huang, Qinghai | 2022 | Effect of occlusion site on the safety and efficacy of intravenous alteplase before endovascular thrombectomy: a prespecified subgroup analysis of DIRECT-MT | RCT |
| 11 | F. N. Alemseged, F. C. Williams, C. Puetz, V. Boulouis, G. Kleinig, T. J. Rocco, A. Wu, T. Y. Shah, D. Arba, F. Kaiser, D. Di Giuliano, F. Morotti, A. Sallustio, F. Dewey, H. M. Bailey, P. O'Brien, B. Sharma, G. Bush, S. Dowling, R. Diomedi, M. Churilov, L. Yan, B. Parsons, M. W. Davis, S. M. Mitchell, P. J. Yassi, N. Campbell, B. C. V. | 2021 | Tenecteplase vs Alteplase before Endovascular Therapy in Basilar Artery Occlusion | RCT |
| 34 | A. J. Barakzie, A. J. G. Cavalcante, F. Nagy, M. Dippel, D. W. J. van der Lugt, A. Roos, Ybwem Majoie, Cblm Ten Cate, H. de Maat, M. P. M. | 2025 | Association of primary and secondary hemostasis biomarkers with acute ischemic stroke outcome in patients undergoing thrombectomy, with or without thrombolytics: post hoc analysis of the Multicenter Randomized Clinical Trial of Endovascular Treatment for Acute Ischemic Stroke in the Netherlands-NO IV | RCT |
| 45 | S. D. Bracard, Xavier Mas, Jean Louis Soudant, Marc Oppenheim, Catherine Moulin, Thierry Guillemin, Francis | 2016 | Mechanical thrombectomy after intravenous alteplase versus alteplase alone after stroke (THRACE): a randomised controlled trial | RCT |
| 73 | V. L. Chalos, N. E. Uyttenboogaart, M. Lingsma, H. F. Mulder, Mjhl Venema, E. Treurniet, K. M. Eshghi, O. van der Worp, H. B. van der Lugt, A. Roos, Ybwem Majoie, Cblm Dippel, D. W. J. Roozenbeek, B. Coutinho, J. M. | 2019 | Endovascular Treatment With or Without Prior Intravenous Alteplase for Acute Ischemic Stroke | RCT |
| 80 | L. W. Chen, Y. Yao, J. Wu, Q. Zhang, G. Xu, S. Yang, P. Zhang, Y. Zhang, L. Li, Z. Xing, P. Shen, H. Shi, H. Liu, J. Xu, C. Wu, P. | 2025 | Effect of Intravenous Alteplase Before Endovascular Thrombectomy on Outcome After Unsuccessful Recanalization in the DIRECT-MT Trial | RCT |
| 108 | Q. W. Z. Deng, L. Liu, Y. K. Zhou, F. Yuan, Z. H. Wang, X. X. Gao, J. Yang, P. F. Zhang, Y. W. Xing, P. F. Li, Z. F. Hong, B. Han, H. X. Shi, H. Z. Shi, H. C. Liu, J. M. Direct Mt Investigators | 2024 | Effect of Time Window on Endovascular Thrombectomy with or without Intravenous Thrombolysis in Acute Ischemic Stroke: Results from DIRECT-MT | RCT |
| 144 | U. K. Fischer, Johannes Strbian, Daniel Eker, Omer Cognard, Christoph Plattner, Patricia S Bütikofer, Lukas Mordasini, Pasquale Deppeler, Sandro Pereira, Vitor M | 2022 | Thrombectomy alone versus intravenous alteplase plus thrombectomy in patients with stroke: an open-label, blinded-outcome, randomised non-inferiority trial | RCT |
| 163 | M. M. Goyal, B. K. Ospel, J. Almekhlafi, M. Zerna, C. Nogueira, R. McTaggart, R. Demchuk, A. M. Poppe, A. Y. Buck, B. Heard, K. Joshi, M. Haussen, D. Cutting, S. Coutts, S. B. Roy, D. Rempel, J. L. Field, T. S. Dowlatshahi, D. Adel, B. V. Swartz, R. Shah, R. Sauvageau, E. Puetz, V. Silver, F. L. Campbell, B. Chapot, R. Tymianski, M. Hill, M. D. | 2025 | Factors Influencing Nerinetide Effect on Clinical Outcome in Patients Without Alteplase Treatment in the ESCAPE-NA1 Trial | RCT |
| 186 | J. W. v. V. Hoving, H. Kappelhof, M. Tolhuisen, M. Treurniet, K. M. LeCouffe, N. E. Rinkel, L. A. Koopman, M. S. Cavalcante, F. Konduri, P. R. van den Wijngaard, I. R. Ghariq, E. Meijer, F. J. A. Coutinho, J. M. Marquering, H. A. Roos, Ybwem Emmer, B. J. Majoie, Cblm | 2023 | Infarct Evolution in Patients with Anterior Circulation Large-Vessel Occlusion Randomized to IV Alteplase and Endovascular Treatment versus Endovascular Treatment Alone | RCT |
| 203 | Z. Y. Z. Jia, Yong Xin Cao, Yue Zhou Zhao, Lin Bo Shi, Hai Bin Zhang, Lei Li, Zi Fu Shen, Hong Jian Lou, Min Zhang, Yong Wei | 2022 | Effect of baseline infarct size on endovascular thrombectomy with or without intravenous alteplase in stroke patients: a subgroup analysis of a randomized trial (DIRECT‐MT) | RCT |
| 234 | R. P. Knapen, F. A. V. Langezaal, L. C. M. Brouwer, J. Majoie, Cblm Emmer, B. J. Vos, J. A. van Doormaal, P. J. Yoo, A. J. Bruggeman, A. A. E. Nijeholt, Gjla van der Leij, C. van Oostenbrugge, R. J. van Zwam, W. H. Schonewille, W. J. Mr Clean Registry Investigators | 2024 | Intravenous Thrombolysis Before Endovascular Treatment in Posterior Circulation Occlusions: A MR CLEAN Registry Study | RCT |
| 247 | N. E. K. LeCouffe, Manon Treurniet, Kilian M Rinkel, Leon A Bruggeman, Agnetha E Berkhemer, Olvert A Wolff, Lennard van Voorst, Henk Tolhuisen, Manon L Dippel, Diederik WJ | 2021 | A randomized trial of intravenous alteplase before endovascular treatment for stroke | RCT |
| 271 | H. Y. Z. Ma, Y. Gao, L. Liu, P. Zhang, L. Xing, P. F. Li, Z. F. Shen, H. J. Zhang, H. J. Zhang, Y. X. Zhang, X. X. Hua, W. L. Zhang, Y. W. Liu, J. M. Yin, C. G. Yang, P. F. | 2023 | Cost-effectiveness of thrombectomy alone versus alteplase before thrombectomy in acute ischemic stroke: results from the DIRECT-MT | RCT |
| 306 | J. Z. Mocco, Osama O von Kummer, Rüdiger Yoo, Albert J Gupta, Rishi Lopes, Demetrius Frei, Don Shownkeen, Harish Budzik, Ron Ajani, Zahra A | 2016 | Aspiration thrombectomy after intravenous alteplase versus intravenous alteplase alone | RCT |
| 311 | K. W. F. Muir, Gary A Messow, Claudia-Martina Ford, Ian Murray, Alicia Clifton, Andrew Brown, Martin M Madigan, Jeremy Lenthall, Rob Robertson, Fergus | 2017 | Endovascular therapy for acute ischaemic stroke: the Pragmatic Ischaemic Stroke Thrombectomy Evaluation (PISTE) randomised, controlled trial | RCT |
| 382 | J. L. G. Saver, Mayank Bonafe, Alain Diener, Hans-Christoph Levy, Elad I Pereira, Vitor M Albers, Gregory W Cognard, Christophe Cohen, David J Hacke, Werner | 2015 | Stent-retriever thrombectomy after intravenous t-PA vs. t-PA alone in stroke | RCT |
| 419 | K. M. Suzuki, Yuji Takeuchi, Masataka Morimoto, Masafumi Kanazawa, Ryuzaburo Takayama, Yohei Kamiya, Yuki Shigeta, Keigo Okubo, Seiji Hayakawa, Mikito | 2021 | Effect of mechanical thrombectomy without vs with intravenous thrombolysis on functional outcome among patients with acute ischemic stroke: the SKIP randomized clinical trial | RCT |
| 453 | K. R. K. van Kranendonk, Manon Bruggeman, Agnetha AE Rinkel, Leon A Treurniet, Kilian M LeCouffe, Natalie Emmer, Bart J Coutinho, Jonathan M Wolff, Lennard van Zwam, Wim H | 2023 | Hemorrhage rates in patients with acute ischemic stroke treated with intravenous alteplase and thrombectomy versus thrombectomy alone | RCT |
| 490 | P. Z. Yang, Yongwei Zhang, Lei Zhang, Yongxin Treurniet, Kilian M Chen, Wenhuo Peng, YA Han, Hongxing Wang, Jiyue Wang, Shouchun | 2020 | Endovascular thrombectomy with or without intravenous alteplase in acute stroke | RCT |
| 517 | W. Q. Zi, Zhongming Li, Fengli Sang, Hongfei Wu, Deping Luo, Weidong Liu, Shuai Yuan, Junjie Song, Jiaxing Shi, Zhonghua | 2021 | Effect of endovascular treatment alone vs intravenous alteplase plus endovascular treatment on functional independence in patients with acute ischemic stroke: the DEVT randomized clinical trial | RCT |
| 206 | M. S. Jones, M. J. Ashley, W. W. Tsimpas, A. | 2018 | Chemical Thrombolysis and Mechanical Thrombectomy for Acute Ischemic Stroke | RCT |

**Table S3.** A list of covariates adjusted for in the analysis of included studies

| Study ID | Confounding Adjustment | Factors adjusted for in the analysis |
| --- | --- | --- |
| Chang (2020) | Yes | Age, race, sex, baseline mRS, collateral grade, discovery to recanalization time, and outside hospital |
| Da Ros (2021) | Yes | Age, vasospasm, DM, ICH, ventilation time, NIHSS score |
| Derraz (2023) | Yes | Age, gender, wake-up stroke, direct admission, medical history (HTN, DM, hypercholesterolemia, smoking, prior stroke, antiplatelet/anticoagulant use, NIHSS score, ASPECTS, prestroke mRS score >1, EVT characteristics (imaging type, occlusion site, time from stroke onset to imaging, and stroke etiology) |
| Di Maria (2018) | Yes | Age, gender, direct admission, medical history (HTN, DM, hypercholesterolemia, smoking, antiplatelet use, anticoagulant use, NIHSS score, ASPECTS, pre-stroke ranking score, occlusion site, favorable collateral, and stroke etiology |
| Dicpinigaitis (2022) | Yes | age, sex, payer status, hospital location and teaching status, admission source, acute stroke severity indices, and comorbid conditions. |
| El Malky (2022) | Yes | NCD |
| Faizy (2022) | Yes | Atrial fibrillation, usage of anticoagulants or antiplatelet drugs, presentation National Institutes of Health Stroke Scale, time from symptom onset to imaging, age, favorable TLC, and favorable pial arterial collateral status on CTA |
| Fang (2022) | Yes | age; sex; blood pressure on admission; smoking and drinking habits; history of taking statins, anticoagulants, and antiplatelet drugs; the CHA2DS2-VASc score; modified Rankin scale (mRS) score; the NIHSS score; ASPECTS or pc-ASPECTS on admission; the history of coronary heart disease, hypertension, diabetes, atrial fibrillation, and ischemic stroke; the location of the occlusion; the TOAST type of stroke; time from onset to perform CT (computed tomography) examination; platelet count; platelet volume; and NLR on admission |
| Ferrigno (2018) | Yes | NCD |
| Gong (2019) | Yes | Age, gender, NIHSS on admission, DM, AFib, HTN, WBC, CRP, D-dimer, neutrophilic granulocytes, time from symptom onset to EVT/vascular recanalization |
| Guedin (2015) | No | - |
| Guo (2024) | Yes | NCD |
| Huu An (2022) | No | - |
| Kaesmacher (2018) | Yes | Age, NIHSS baseline |
| Kurminas (2020) | No | - |
| Le Floch (2023) | Yes | NCD |
| Maier (2017) | No | - |
| Molad (2023) | No | - |
| Seetge (2024) | Yes | Age, pre-mRS score, admission, NIHSS score, mASPECTS, and mCTA collateral score |
| Smith (2006) | No | - |
| Tong (2021) | Yes | Age, gender, HTN, DM, dyslipidemia, CAD, AFib, prior ischemic stroke, smoking, admission mode, onset-to-door time, SBP, NIHSS score, ASPECTS, occlusion site, stroke subtype, anesthesia type |
| Wang (2017) | Yes | Age, gender, prior stroke, baseline NIHSS score, ASPECTS, mRS score, stroke etiology, occlusion site, time from onset to door, collateral status (ASITN/SIR) |
| Weber (2017) | No | - |
| Smith (2022) | Yes | NCD |
| Ahmed (2021) | Yes | NCD |
| Casetta (2019) | Yes | age, sex, history of diabetes, atrial fibrillation, hypertension, previous stroke or transient ischemic attack in the previous three months, the presence of carotid stenosis >70%, baseline NIHSS score, wake-up stroke, onset to door time, onset to groin puncture time, site of occlusion, and ASPECTS score |
| Chalos (2019) | Yes | age, baseline NIHSS, history of diabetes mellitus, pre-stroke mRS, prior use of anticoagulant medication, onsetto-first-noncontrast-computed-tomography time, center (in case of sufficient [≥1] outcome events), and additional baseline imbalances (P<0.05) in the patients’ medical histories |
| Geng (2021) | Yes | Age, gender, ethnicity, BMI, SBP, DBP, pulse, TOAST, large-artery atherosclerosis, cardioembolism, small-artery occlusion, stroke of other determined cause, way to hospital (EMS, interhospital transfer, personal transport, NIHSS, mRS), hospital level, hospital region, |
| Leker (2018) | Yes | admission NIHSS (per point), age (per year), gender, time to endovascular treatment, and stroke subtype to |
| Minnerup (2016) | Yes | age, sex, NIHSS upon admission, comorbidities, stroke etiology and most proximal occlusion location |
| Park (2017) | Yes | SHT, initial random glucose, OtEVT, stroke history, anticoagulant use, onset-to-hospital arrival, onset-to-EVT, hospital-to-EVT |

NCD: not clearly described; DM: diabetes mellitus; EVT: endovascular therapy; ICH: intracranial hemorrhage; HTN: hypertension; mRS: modified Rankin score; SBP: systolic blood pressure; DBP: diastolic blood pressure; EMS: emergency medical service; AFib: atrial fibrillation; CAD: coronary artery disease; NIHSS: National Institutes of Health Stroke Scale; ASPECTS: Alberta Stroke Program Early CT Score.
